# The effect of a systematic multi-dimensional assessment in severe uncontrolled asthma: a literature review and protocol for an investigator-initiated, open-label, randomized-controlled trial (EXACT@home study)

**DOI:** 10.1186/s12890-025-03646-5

**Published:** 2025-05-17

**Authors:** L. Bult, G. J. Braunstahl, J. G. J. V. Aerts, D. Bänffer, J. S. J. A. van Campen, M. S. van Daalen, Y. van Dooren, U. Flanders, E. S. Geurts, P. P. Hekking, R. Heller - Baan, M. J. A. Jans, J. H. Kappen, R. C. A. Mies, B. Oppedijk, M. de la Roij - Hartmans, S. Van der Sar - Van der Brugge, Y. Türk, E. Vis, R. Wolters, E. C. Vasbinder, J. C. C. M. in ‘t Veen

**Affiliations:** 1 Department of Pulmonary Medicine, Franciscus Gasthuis and Vlietland, Rotterdam, The Netherlands; 2 Department of Pulmonary Medicine, Erasmus MC University Medical Centre, Rotterdam, The Netherlands; 3 Pulmonary Rehabilitation Center Revant, Breda, the Netherlands; 4 Department of Pulmonary Medicine, Haaglanden Medical Center, The Hague, The Netherlands; 5 Department of Hospital Pharmacy, Franciscus Gasthuis and Vlietland, Rotterdam, the Netherlands; 6 Department of Pulmonary Medicine, Groene Hart Hospital, Gouda, The Netherlands; 7 Department of Pulmonary Medicine, Elisabeth-TweeSteden Hospital, Tilburg, The Netherlands; 8 Department of Physiotherapy, Franciscus Gasthuis and Vlietland, Rotterdam, The Netherlands; 9 Department of Pulmonary Medicine, Ikazia Hospital, Rotterdam, The Netherlands; 10 Department of Pulmonary Medicine, Beatrix Hospital, Gorinchem, The Netherlands; 11 National Heart and Lung Institute, Imperial College London, UK Centre in Allergic Mechanisms of Asthma, London, Asthma UK; 12 Department of Pulmonary Medicine, Maasstad Hospital, Rotterdam, The Netherlands; 13 Department of Pulmonary Medicine, Bravis Hospital, Bergen Op Zoom & Roosendaal, The Netherlands; 14 Department of Pulmonary Medicine, Amphia Hospital, Breda, The Netherlands; 15 Pulmonary Rehabilitation Center, Dutch Asthma Center Davos (NAD), Davos, Switzerland; 16 Department of Pulmonary Medicine, Admiraal de Ruyter Hospital, Goes, The Netherlands; 17 STZ Center of Excellence for Asthma, COPD, Respiratory Allergy and Occupational Lung Diseases, Department of Pulmonology, Franciscus Gasthuis en Vlietland, Kleiweg 500, 3045 PM Rotterdam, The Netherlands

**Keywords:** Systematic assessment, Treatable traits, Difficult-to-treat asthma, Severe asthma, EHealth

## Abstract

**Introduction:**

Severe asthma affects 3.6% of the asthma population, in which patients are uncontrolled despite optimal drug therapy and management of treatable traits. These patients are eligible for treatment with biologicals, which provide significant benefits but are costly and need precise indication. However, identifying all individual treatable traits before diagnosing severe asthma is challenging. A systematic multi-dimensional assessment may help identify and address these hidden traits, resulting in tailored treatment and reducing the number of unnecessary biological prescriptions.

**Methods:**

A literature review was conducted to address the knowledge gap on the effectiveness and added value of a systematic assessment and treatment in difficult-to-treat or severe asthma, followed by an outline of a study protocol to implement this in patients diagnosed with severe asthma.

**Results:**

The literature review revealed limited evidence on the effectiveness of systematic assessments in difficult-to-treat or severe asthma, largely due to the use of different study methods and outcome measures. Notably, only one of the selected articles employed a randomized controlled design. To address this gap, the EXpert Asthma Copd Trajectory with digital support (EXACT@home) study was proposed, which aims to improve the assessment and treatment of treatable traits in severe asthma before (re)considering treatment with biologicals. This study uses a prospective, open label, randomized controlled trial design with the primary aim of reducing biological prescriptions. Patients are eligible for inclusion if they have previously been diagnosed with severe uncontrolled asthma with an indication for treatment with biologicals. The intervention arm undergoes a 6-week systematic assessment program targeting treatable traits followed by tailored treatment, while the control arm directly receives treatment with biologicals. Both arms are followed for 12 months with secondary outcomes including asthma control, quality of life and exacerbation frequency.

**Discussion:**

Difficult-to-treat or severe asthma requires tailored treatments based on individual treatable traits, but challenges remain in accurately identifying these traits. Existing literature highlights the beneficial effects of systematic assessments, but conclusive evidence is lacking. The EXACT@home study aims to provide high quality evidence on the effectiveness of such an assessment in the management of severe uncontrolled asthma, addressing a gap in the current literature.

**Trial registration:**

NCT05831566 (Clinicaltrials.gov), registered at 14–04-2023.

*Protocol version:* version 6, date 27–03-2024.

**Supplementary Information:**

The online version contains supplementary material available at 10.1186/s12890-025-03646-5.

## Introduction

Asthma is a common multifactorial disease characterized by chronic inflammation and airway hyperresponsiveness to direct or indirect stimuli, resulting in intermittent symptoms like cough, wheezing, shortness of breath and/or chest tightness [1].

In the Netherlands, more than 500,000 patients have been diagnosed with asthma. Of these patients 17% have difficult-to-treat asthma that is uncontrolled despite optimal use of inhaled medication and/or maintenance therapy with oral corticosteroids (mOCS). This is often due to the presence of treatable traits, which are factors that contribute to the disease severity [2]. Within the field of difficult-to-treat asthma, severe asthma is a distinct subpopulation, affecting 3.6% of the total asthma population [2]. Severe asthma is defined as asthma that remains uncontrolled despite adherence to optimised high-intensity therapy and optimal treatment of treatable traits [3]. Treatable traits can be categorized into pulmonary factors (e.g. eosinophilia, allergies, airway obstruction and infections), non-pulmonary factors such as comorbidities (e.g. obesity, depression, vocal cord dysfunction, rhinosinusitis, obstructive sleep apnea syndrome and gastroesophageal reflux), behavioral aspects (e.g. adherence, inhaler technique, physical activity, dysfunctional breathing, symptom perception, smoking) and environmental influences (e.g. social network, pollution) [4–6]. Patients with difficult-to-treat asthma have similar clinical presentation, but possess unique compositions of underlying treatable traits.

Severe asthma is considered an ‘orphan disease’, but it is responsible for a high disease and healthcare burden [7–9]. Treatment with biologicals have fundamentally changed the care of patients with severe asthma, resulting in significant improvement in asthma control, lung function and quality of life and decrease of exacerbation frequency and use of mOCS [10–12]. Nevertheless, hidden, untreated treatable traits at the start of treatment with biologicals may lead to suboptimal treatment outcomes and a delay in the initiation of appropriate treatment. Addressing these hidden traits through systematic assessment first could be sufficient, potentially eliminating the need for biologicals.

The Centre of Excellence for Severe Asthma, Franciscus Gasthuis and Vlietland (FGV), Rotterdam, the Netherlands, provides such a validated tertiary assessment, known as EXACT (EXpert Asthma Copd Trajectory) [13], designed to systematically assess and manage these traits in patients with difficult-to-treat asthma. This assessment includes a comprehensive systematic assessment of treatable traits [14], which is performed by a respiratory nurse and includes disease-related patient-reported outcome measures (PROMs). The assessment also includes a comprehensive spirometry and oscillometry measurement, one week of activity monitoring and a consultation with a physiotherapist. Finally, a meeting is held between the respiratory nurse and the pulmonologist to discuss the patient’s case in detail so that the pulmonologist can decide on a personalized treatment plan.

Given the potential significance of systematically assessing and managing treatable traits in uncontrolled asthma, we conducted a literature review to assess the current evidence regarding the effectiveness of these assessment programs. Next, we developed a study protocol to implement an in-depth assessment building upon the existing EXACT program, optimised with digital home monitoring and a wide range of PROMs, for patients diagnosed with severe asthma. The primary aim of this study is to uncover potentially hidden treatable traits and reduce unnecessary prescription of expensive biologicals.

## Methods

### Search strategy literature review

A Pubmed search was conducted based on the following research question: ‘Does a multidimensional systematic assessment of treatable traits contribute to optimized and personalised treatment in difficult-to-treat or severe asthma?’ Two authors (LB and JV) independently assessed the identified articles based on the title and abstract to determine their inclusion in this review. Disagreements were resolved by seeking the opinion of a third author (GB). Only original English-language studies were considered for inclusion. Further information about the PubMed search and the selection process can be found in the Suppl.1.

### Assessment of evidence

The primary outcome was the effectiveness of a systematic assessment program, as indicated by clinical parameters such as the Asthma Control Questionnaire (ACQ), the Asthma Quality of Life Questionnaire (AQLQ), healthcare utilisation (HCU), pulmonary function (forced expiratory volume in 1 s (FEV1)), exacerbation frequency and usage of mOCS. Each study underwent an evaluation based on multiple criteria to assess the evidence supporting the research question. The strength of the study design was assessed using a star rating system: one star for retrospective studies, two stars for prospective observational studies, and three stars for randomised controlled trials.

## Results

A PubMed search was conducted using search terms related to the research question (suppl. 1). This yielded a total of 150 articles. Following a selection process based on the title and abstract, eight articles were suitable for further evaluation (Table 1). Only one of these selected articles used a randomized controlled design, while the remaining seven used a prospective observational approach.

**Table 1:** Overview of trials investigating the effect of a (multidisciplinary) systematic assessment in difficult-to-treat or severe asthma

References	Study design	Strenght	N	Study population	Study description	Clinical outcomes
Irwin et al. 1993 [15]	Prospective, observational	★★★	42	Adult patients with difficult asthma referred for systematic assessment in Massachusetts between January 5, 1982 to October 1, 1990	▪ Systematic assessment: ▪ Diagnostic confirmation ▪ Identify poor adherence ▪ Identify poor inhaler technique ▪ Address comorbidities and contributing factors (sinusitis, medication/food additive sensitivity, environment/work-related triggers, ABPA) ▪ Assessment of why asthma was difficult to control and identification of the most helpful therapeutic interventions	3.5 years follow-up: ▪ 74% were no longer DTC after systematic assessment ▪ Improvement more likely if GERD was a factor ▪ Non-adherence most likely reason for maintaining DTCA
Heaney et al. 2003 [16]	Prospective, observational	★★★	73	Adult patients with uncontrolled asthma referred for systematic assessment in Belfast, Ireland	▪ Systematic assessment: ▪ Diagnostic confirmation ▪ Inflammatory phenotyping: blood tests; induced sputum analysis ▪ Comorbidity and contributory factor detection: medical history; psychological status; skin prick testing; assessment inhaler technique; psychiatric assessment; HR-CT thorax; pulmonary function tests; ENT examination and/or imaging; oesophageal pH monitoring ▪ Self-management plan ▪ Peak flow record and asthma diary ▪ Outcome measure(s): AQLQ	▪ 39 responded to intervention and 34 had TRA ▪ High and similar prevalence of comorbidities in both groups 12 months follow-up: ▪ Significant AQLQ improvement in therapy-responsive asthma (3.2 to 4.4), but not in TRA (3.3 to 3.6)
Gibeon et al. 2015 [17]	Prospective, observational	★★★	346	Adult patients with difficult-to-treat asthma referred to one of the 11 centers within the National Registry for UK Difficult Asthma Services between April 2009 and December 2010	▪ Systematic assessment: ▪ Diagnostic confirmation ▪ Inflammatory phenotyping: blood tests ▪ Comorbidity and contributory factor detection: e.g. medical history; pulmonary function tests; allergy assessment ▪ Outcome measure(s): ACQ, HCU, FEV1, (m)OCS	Median 286 days follow-up: ▪ Significant improvement in AQLQ and ACQ (3.6 to 3.0, 3.4 to 2.8) ▪ Significant reduction in HCU ▪ No difference in required mOCS, but reduced steroid dose and short-burst steroids
Van der Meer et al. 2016 [18]	Prospective, observational	★★★	40	Adult patients with uncontrolled asthma referred for systematic assessment in Leeuwarden, The Netherlands, between June 2013 and June 2014	▪ Systematic assessment: ▪ Diagnostic confirmation ▪ Inflammatory phenotyping: blood tests; induced sputum analysis; FeNO ▪ Comorbidity and contributory factor detection: medical history; psychological status; HR-CT thorax; pulmonary function tests; adherence and inhaler technique; ENT examination/imaging; 6MWD ▪ Outcome measure(s): ACQ, AQLQ, HCU, exacerbations	12 months follow-up: ▪ Significant improvement in ACQ and AQLQ (2.6 to 1.8 and 4.8 to 5.4) ▪ Significant reduction exacerbation frequency (≥ 2) and HCU
Tay et al. 2017 [19]	Prospective, observational	★★★	65	Adult patients with difficult asthma referred for systematic assessment in Melbourne, Australia, between June 2014 and March 2016 1.5% were on biologicals at baseline (anti-IL5)	▪ Systematic assessment: ▪ Diagnostic confirmation ▪ Inflammatory phenotyping ▪ Comorbidity and contributory factor detection: obesity; allergic rhinitis; CRS; gastroesophageal reflux; obstructive sleep apnoea; anxiety or depression; dysfunctional breathing and vocal cord dysfunction; history of potential aggravating factors (e.g. environmental exposure); poor medication adherence and inhaler technique ▪ 15 patients used a smartinhaler measuring adherence ▪ Outcome measure(s): ACT, AQLQ, exacerbations	▪ Median of 3 comorbidities, and 3 comorbidity interventions 6 months follow-up: ▪ Overall improvements in ACT and AQLQ (14 to 16 and 4.3 to 4.7) ▪ Significant reduction exacerbation frequency (2 to 0) ▪ Median medication adherence rate measured with Smartinhaler (n = 15): 87%
Denton et al. 2019 [20]	Prospective, observational	★★★	161	Adult patients with difficult asthma referred for systematic assessment in Melbourne, Australia, between June 1, 2014, and December 31, 2017 6.0% were on biologicals at baseline, and 7.0% commenced treatment during the assessment period	▪ Systematic assessment: ▪ Diagnostic confirmation ▪ Inflammatory phenotyping ▪ Identify poor adherence (41% with electronic adherence monitor) ▪ Identify poor inhaler technique ▪ Comorbidity and contributory factor detection: adherence and inhaler technique; allergic rhinitis; CRS; OSA; GERD; dysfunctional breathing; VCD; psychiatric history ▪ Outcome measure(s): ACT, AQLQ, FEV1, mOCS, exacerbations	6 months follow-up: ▪ 87% improved in at least 1 domain ▪ 64% had a reduction in exacerbations, 54% achieved MID for ACQ and AQLQ, and 40% increased FEV1 ≥ 100 mL ▪ mOCS burden halved, comparable to results achieved with monoclonal biologicals
McDonald et al. 2020 [21]	Randomized, open-label, cross-sectional	★★★	55	Adult patients with severe asthma treated at a severe asthma clinic in Newcastle, Australia	▪ Intervention: 16-week treatment program with comprehensive multidimensional assessment across pulmonary, extrapulmonary, and behavioural/lifestyle domains, followed by targeted treatment ▪ Usual care: Treatment according to best available evidence by the pulmonologist ▪ Outcome measure(s): ACQ, AQLQ	▪ Mean of 10.44 traits per person: 3.01 pulmonary traits, 4.85 extrapulmonary traits, 2.58 behavioural/lifestyle traits 4 months follow-up: ▪ Individualised treatment targeting traits was feasible and significantly improved AQLQ (0.86 units) and ACQ (0.73 units)
Lin et al. 2023 [22]	Prospective, observational	★★★	241	Adult patients with difficult asthma referred for systematic assessment in Melbourne, Australia, between June 2014 and April 2022 7.5% were on biologicals at baseline	▪ Systematic assessment: ▪ Diagnostic confirmation ▪ Inflammatory phenotyping ▪ Comorbidity and contributory factor detection: obesity; allergic rhinitis; CRS; gastroesophageal reflux; obstructive sleep apnea; anxiety or depression; dysfunctional breathing and vocal cord dysfunction; history of potential aggravating factors (e.g. environmental exposure); poor medication adherence and inhaler technique ▪ Latent class analysis performed using 12 traits ▪ Outcome measure(s): ACT, AQLQ, FEV1, mOCS, exacerbations	▪ Worse baseline ACQ and AQLQ in non-airway-centric profiles 6 month follow-up: ▪ Overall improvements across all outcomes: ACQ (0.5 units), AQLQ (0.6 units), FEV1 (3.8%), mOCS dose (3.3 mg), exacerbation frequency (1.4 exacerbations) ▪ Airway-centric (early-onset with allergic rhinitis or adult onset with eosinophilia/CRS): more FEV1 improvement (trend) ▪ Non-airway-centric (comorbid, psychosocial or multi-domain): greater reduction in exacerbations

*Abbreviations: TR* therapy resistant asthma*, ENT* ear nose throat, *HCU* healthcare utilization, *FEV1* forced expiratory volume in 1 s, *(m)OCS* (maintenance) oral corticosteroids, *CRS* chronic rhinosinusitis, *MID* minimal important difference (MID ACQ and AQLQ is 0.5)

This selection consisted of articles with a wide range of study designs in terms of the method, content and duration of the assessment and follow up. In addition, populations and outcome measures differed (Table 1). However, all articles [15–20, 22, 23] share three components: (1) confirmation of the asthma diagnosis, (2) assessment of the inflammatory phenotype, and (3) assessment of contributing factors and/or comorbidities. The studies by Tay et al. [19] and Lin et al. [22] used the same systematic assessment protocol and were conducted in the same hospital, but involved distinct study populations.

The study by McDonald et al. [21] was the only randomized controlled trial, comparing standard care with a systematic assessment. This study concluded that individualized treatment based on treatable traits was feasible and resulted in improvements in asthma quality of life (AQLQ) and asthma control (ACQ). Of all selected articles, six demonstrated significant improvements in asthma control (ACQ or ACT) and/or asthma quality of life (AQLQ) [17–22]. Conversely, Heaney et al. [16] reported that targeted treatment of identified comorbidities had minimal impact on quality of life in individuals with therapy-resistant asthma (TRA), a condition equivalent to severe uncontrolled asthma. In addition, three articles documented a reduction in mOCS burden [17, 20, 22], with Denton et al. [20] demonstrating a reduction in mOCS burden comparable to that achieved with biological therapy. Moreover, five articles demonstrated reduced exacerbation rates [17–20, 22], two of which noted significant lung function improvements [20, 22], while two others observed enhanced health care utilization (HCU) [17, 18].

Three articles describe a small percentage of patients who were already using asthma biologicals at baseline and/or during assessment, which might have influenced the results [19, 20, 22]. However, Denton et al. [20] concluded that the significant improvement in asthma outcomes remained statistically and clinically significant after excluding patients commenced on biologicals.

Furthermore, earlier research by Irwin et al. [15] did not specifically examine improvements in the aforementioned clinical outcome parameters but emphasized the potential effectiveness of addressing gastroesophageal reflux disease (GERD) and enhancing adherence as key strategies for managing difficult-to-control asthma.

Finally, a few studies explored the use of eHealth to assess treatable traits in a home setting. Tay et al. [19] (investigated adherence in a small subgroup of 15 patients using a smart inhaler and reported an adherence rate of 87%. Similarly, Denton et al. [20] used an electronic adherence monitor in 41% of patients but did not report adherence outcomes. In contrast, Heaney et al. [16] included peak flow measurements as part of their systematic assessment. However, the results of these measurements were not disclosed in the article.

## Conclusion of the literature review

The existing literature underscores the potential benefits of systematic assessment, yet the overall evidence remains limited and inconclusive. Most studies varied in terms of study population, methods, outcome measures, content of the systematic assessment, and follow-up duration. Consequently, it is clear that a larger randomized controlled trial is necessary to more effectively assess the benefits of a comprehensive systematic assessment.

Hence, we propose a study protocol for a randomized controlled trial investigating an in-depth multidimensional systematic assessment of hidden treatable traits, extended to include digital home monitoring and multiple PROMs, in patients with severe uncontrolled asthma who have previously undergone a conventional assessment: the EXACT supported by eHealth, or EXACT@home for short. This study aims to further improve the systematic assessment and management of patients with severe asthma, thereby establishing a more patient-centred approach and optimising the indication for treatment with biologicals.

### Study protocol: EXpert Asthma Copd Trajectory with digital support (EXACT@home)

The EXACT@home study was initiated to provide evidence on the added value of a comprehensive, multidimensional, and systematic assessment of hidden treatable traits, including digital home monitoring and multiple PROMs, in severe, uncontrolled asthma. The aim is to enhance personalized treatment strategies and optimize the indication for treatment with biologicals. The primary objective is to determine whether the EXACT@home systematic assessment program reduces the proportion of patients requiring therapy with biologicals after six months of follow-up. Secondary objectives include assessing the proportion of patients treated with biologicals after 12 months and evaluating changes in asthma control, quality of life, and exacerbation frequency over the same period as a result of the program.

### Study design

The EXACT@home study is an investigator-initiated, prospective, open-label, randomized controlled trial with a superiority design. The study will be conducted at the Centre of Excellence for Severe Asthma at Franciscus Gasthuis & Vlietland (FGV), with patients recruited from this hospital and its affiliated hospitals. The study has an open-label design to determine the effect of the EXACT@home program in a real-life situation.

**Fig. 1:**
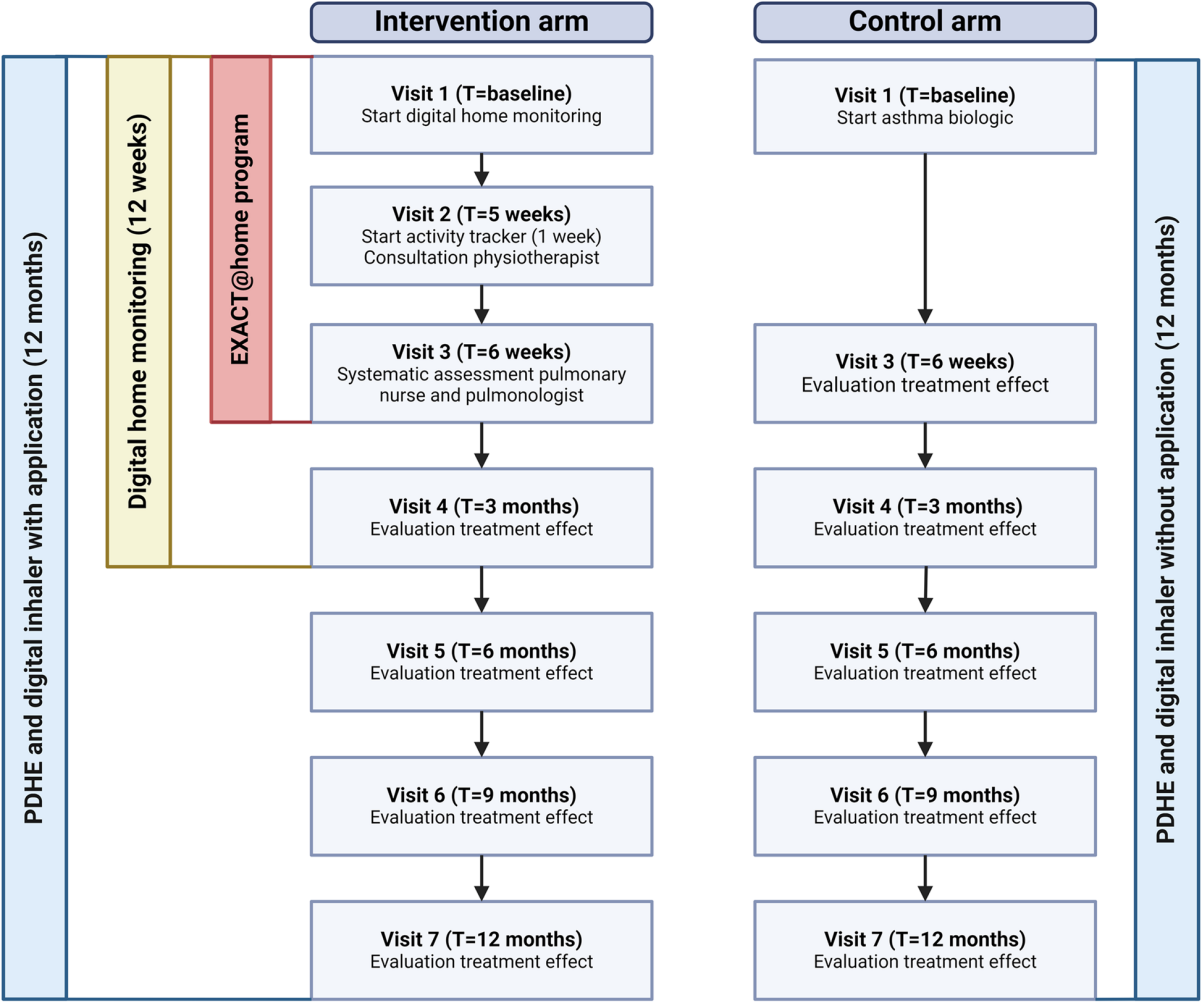
EXACT@home study design. The EXACT@home study consists of 7 visits for participants in the intervention arm and 6 visits for those in the control arm. The systematic assessment program for the intervention arm spans visits 1 to 3 and lasts for 6 weeks. From visit 1 to visit 4 (12 weeks), patients in the intervention arm are monitored at home using a portable spirometer, FeNO meter (for a subgroup), and an activity tracker (Corsano). Additionally, they use a second activity tracker (MoveMonitor) for one week between visits 2 and 3. From visit 1 to visit 7 (12 months), they also use the PDHE and a digital inhaler with the application providing feedback and reminders. In the control arm, patients use the PDHE and digital inhaler without the application for 12 months

Eligible patients diagnosed with severe asthma and recommended for treatment with biologicals are randomly assigned in a 1:1 ratio to the control or intervention arm. The intervention arm undergoes a 6-week EXACT@home program that assesses and targets treatable traits before a treatment decision is made, consisting of biologicals or treatment of identified treatable traits. The control arm directly receives standard of care (considered the gold standard) consisting of treatment with a biological. The follow-up period will be 12 months. The study outline is presented in Fig. 1. Ethics approval for the EXACT@home study was provided by the Medical research Ethics Committees United (NL79996.100.22). Approval by the Institutional Research Board and Board of Directors of the FGV hospital was also provided. The study is registered at clinicaltrials.gov (NCT05831566).

### Study population

Patients aged ≥ 18 years with severe uncontrolled asthma who were eligible for treatment with biologicals according to the regional asthma multi-disciplinary consultation (MDC) organized by the Centre of Excellence for Severe Asthma, FGV are included by the coordinating investigator. The inclusion and exclusion criteria are shown in Table 2. Thirteen affiliated hospitals (Erasmus Medical Centre (ErasmusMC), Haaglanden MC, Maasstad-, Groene Hart-, Ikazia-, Albert Schweitzer-, Van Weel Bethesda-, Admiraal de Ruyter-, Amphia-, ZorgSaam-, Tweesteden-, Bravis- Spijkenisse- and Beatrix hospital) in the southwest region of the Netherlands contribute to this regional asthma MDC. The MDC is used to diagnose severe asthma and make recommendations for biologicals in patients who have previously undergone a conventional assessment at the local affiliated hospital. Currently considered the gold standard, the MDC has the potential to reduce hospital admissions and asthma exacerbations, thereby improving the overall quality of life for people with severe asthma [24]. However, the complexity and heterogeneity of difficult-to-treat asthma often makes it challenging to have a complete picture of a patient and their individual treatable traits before diagnosing severe asthma and initiating a treatment with biologicals. The EXACT@home study aims to further improve the assessment of treatable traits and if necessary reconsider treatment with biologicals. Eligible patients from the local affiliated hospitals are referred to the FGV hospital if they meet the inclusion criteria, do not meet the exclusion criteria (Table 2) and express an interest in participating in the EXACT@home study.

**Table 2:** In- and exclusion criteria

**Inclusion criteria**
• Age ≥ 18 years • Confirmed diagnosis of asthma according to asthma guidelines [1, 25, 26] • Diagnosis of severe, uncontrolled asthma with eligibility for treatment with specific asthma biologicals (omalizumab, mepolizumab, benralizumab, reslizumab, dupilumab, tezepelumab) as determined at the regional asthma MDC according to asthma guidelines [1, 27] • Previously prescribed asthma biologicals must have been discontinued ≥ 4 times the elimination half-life of the specific biological • Relative clinical stability. A minimum of 2 weeks should have passed since the onset of asthma exacerbation and/or lower respiratory tract infection requiring treatment with prednisolone and/or antibiotics
**Exclusion criteria**
• Primary diagnosis of COPD • History of cancer, including: - Current basal cell carcinoma, localised squamous cell carcinoma of the skin, or carcinoma in situ of the cervix. Patients are eligible if curative therapy was completed at least 12 months prior to study entry - Current other malignancies. Patients are eligible if curative treatment was completed at least 5 years before the start of the trial • Inability to read and understand the Dutch language adequately • Inability to participate in a remote monitoring and coaching program using a smartphone • Inability to participate in physical activity (e.g. physical disability) • Current pregnancy • Currently breastfeeding • A liaison with the coordinating or (principal) investigator, which could likely influence the decision to participate in this study voluntarily (in concordance with the WMO – article 5) If a participant becomes pregnant or starts breastfeeding during the study, they will discontinue their participation. Nonetheless, data collected before discontinuation will be included in the final analysis, and the selected treatment will be continued despite the participant’s withdrawal

### Study procedures

The EXACT@home study includes a 12-month follow-up period, with 7 study visits for the intervention arm and 6 for the control arm (Fig. 1). During the initial visit, patients are randomly assigned to either arm.

Both arms complete a variety of PROMs addressing different treatable traits (Table 3). In addition, the intervention arm starts utilizing digital tools for home monitoring of treatable traits, which include the following 5 digital devices and/or mobile applications (Table 4):

1. Gezondheidsmeter, Curavista®: a Personalized Digital Healthcare Environment (PDHE) application including the following features: an information desk providing disease specifics and medication guidance (including inhaler technique), a personalized asthma action plan detailing exacerbation management, options for eConsultation with healthcare providers, an online diary for symptom tracking, PROMs for evaluation, and the capacity to measure lung function and Fractional exhaled Nitric Oxide (FeNO), along with the ability to view these results (Suppl. 3). The PDHE thereby helps to improve self-management skills and promote behavioral change [28, 29].
2. Spirobank Oxi, MIR®: a portable spirometer, integrated with the PDHE, enabling patients to measure their lung function in various settings. This facilitates the identification of dysfunctional breathing patterns and triggers of airway inflammation [30–34].
3. Cardiowatch 327–2, Corsano®: A wrist-worn activity monitor, designed as a wristband and equipped with its own application, tracking physical activity, heart rate, respiratory rate, oxygen saturation, temperature, and sleep patterns. It, among others, serves as a guidance tool to enhance physical activity levels [35].
4. BF-Digihaler-DS, Teva®: A digital dry powder inhaler (DPI) containing 160/4.5 µg budesonide/formoterol, integrated with its own application, monitoring adherence and inhaler technique and providing reminders and feedback for improvement [36, 37].

**Table 3:** PROMs as part of the EXACT@home multi-dimensional systematic assessment of treatable traits

PROM	Abbreviation	Function
Nijmegen Clinical Screening Instrument [38]	NCSI	Used to gain insight into the nature and severity of the problems a patient experiences when living with a chronic condition
Test of Adherence to Inhalers [39]	TAI	Identifies patients with low adherence, determines the degree of adherence and gives an idea of the type or pattern of non-compliance
Marshall questionnaire [40]	Marshall	Measures self-reported weekly physical activity
Epworth Sleepiness Scale [41]	ESS	Measures self-reported daytime sleepiness
Patient Activation Meassure [42]	PAM	Measures self-reported knowledge, skills and confidence in the ability to self-manage a chronic condition. Aims to gain insight in the ability of the patient to improve their health status
(modified) Medical Research Council dyspnea scale [43]	(m)MRC	A dyspnea scale, as a measure of disability in patients with respiratory disabilities
Nijmegen Questionnaire [44]	NQ	Assesses the possibility of a diagnosis of hyperventilation syndrome
Hospital Anxiety and Depression scale [45]	HADS	Measures the level of anxiety and depression

**Table 4:**
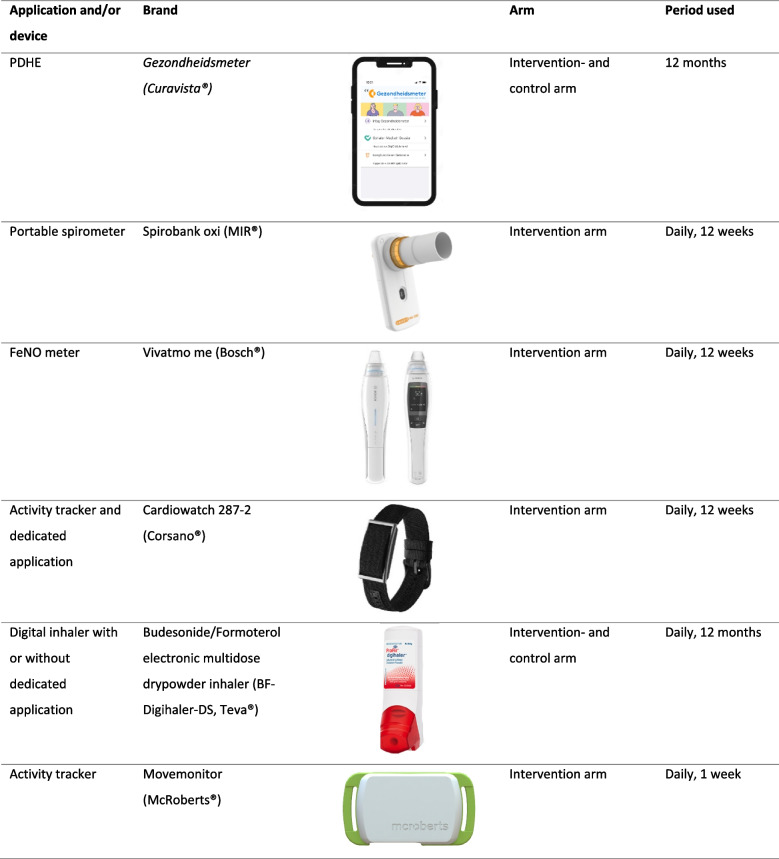
Digital techniques as part of the EXACT@home multi-dimensional systematic assessment of treatable traits

At visit 2, patients begin using a triaxial activity tracker (MoveMonitor, McRoberts®) for one week. Worn as a strap on the lower back, this device measures posture and detects movement (Table 4). Patients also complete the Nijmegen Screening Clinical Instrument (NCSI), a comprehensive PROM that provides insights into the nature and severity of issues related to chronic conditions like asthma [38] (Suppl. 4). Additionally, they fill out the Patient Activation Measure (PAM), which assesses their knowledge, skills, and confidence in managing their condition, enabling tailored care [42] (Table 3). Patients undergo comprehensive spirometry and oscillometry measurements and are assessed by a physiotherapist, who observes their breathing pattern and conducts tests such as the 6-Minute Walk Test (6MWT) [46] and the Short Physical Performance Battery (SPPB) [47]. Based on these evaluations, the physiotherapist provides recommendations regarding the initiation of rehabilitation. During visit 3 a systematic assessment of treatable traits is performed by the respiratory nurse and the pulmonologist according to the alphabet described by Honkoop et al. [14] (Suppl. 2), incorporating the results of the assessment by the physiotherapist, the spirometry and oscillometry measurements, the Patient Reported Outcome Measures (PROMs) and the digital home monitoring. If treatable traits are identified (e.g. non-adherence and physical inactivity), treatment for these are initiated. Otherwise, if no treatable traits are identified, or if the disease remains uncontrolled after fully addressing identified traits, treatment with biologicals will be initiated as decided during the MDC. In contrast, patients in the control arm directly receive treatment with biologicals at visit 1 as decided during the MDC. Patients in the control arm also use the PDHE to complete PROMs and the digital inhaler to measure adherence after the initiation of treatment with biologicals. However, unlike the intervention arm, the digital inhaler in the control arm is not connected to an application that provides reminders and feedback. Both patients in the intervention and control arm are required to switch their inhaled corticosteroids/long-acting beta agonist (ICS/LABA) to the BF-digihaler-DS at the time of inclusion. If the patient is unable to cope with the device or cannot tolerate budesonide/formoterol, they will be switched to an alternative first-line treatment in accordance with the Global Initiative for Asthma (GINA) guidelines [1]. This adjustment does not lead to exclusion from the study, given the primary intention-to-treat design.

**Fig. 2:**
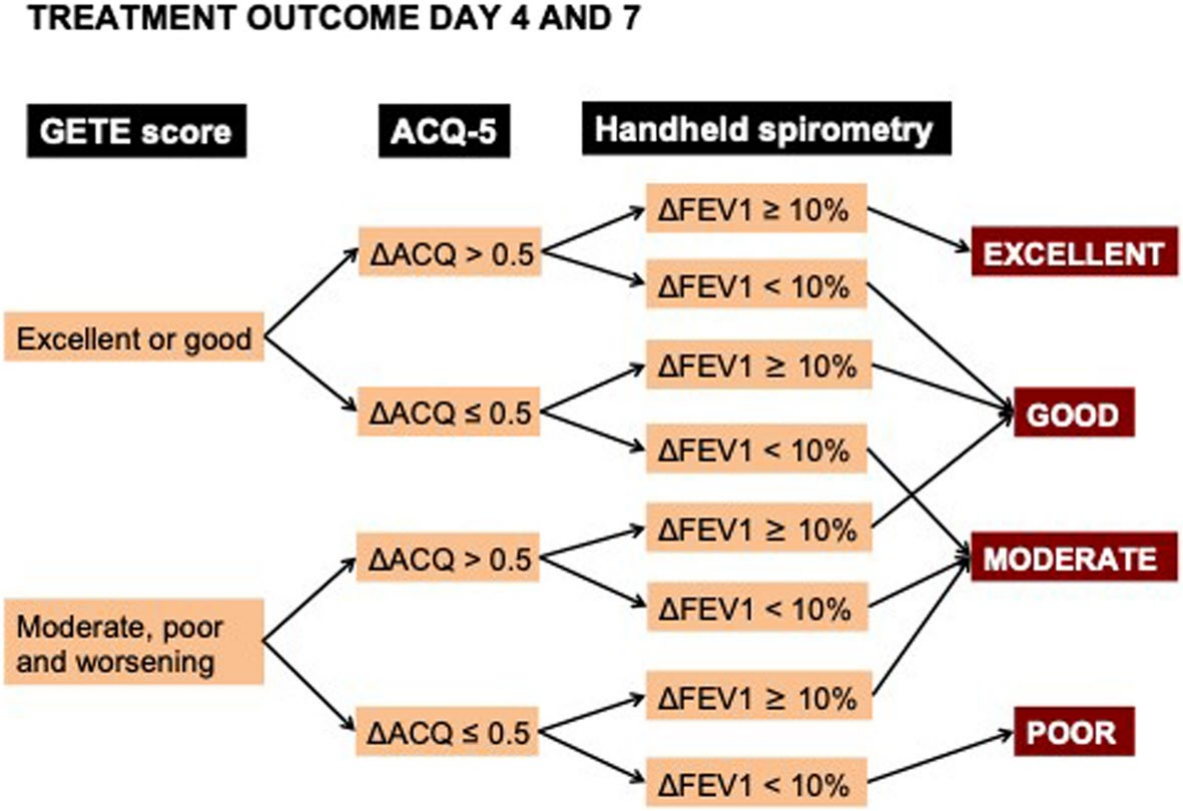
Algorithm guiding treatment in both the intervention- and control arm. Abbreviations: GETE, global evaluation of treatment effectiveness; ACQ, asthma control questionnaire; FEV1, forced expiratory volume in 1 s; Δ >, improvement; Δ <, worsening. *This flowchart is also being utilized in the ExCluSie-F study conducted by Redel *et al*. (NCT05304039)*

In both arms, spirometry and oscillometry with reversibility, along with a FeNO measurement, are conducted at visit 3. Regular spirometry and FeNO measurements are then performed at visits 5 and 7. Additionally, the ACQ and Global Evaluation of Treatment Effectiveness (GETE) [48] are completed at every visit in both arms. The GETE provides a global assessment of treatment efficacy by both clinicians and patients, with scores such as excellent, good, moderate, poor, or worsening. Starting at visit 3 in the control arm and visit 4 in the intervention arm, a flowchart incorporating ACQ, FEV1, and GETE is used at each visit to guide decisions regarding the continuation of treatments (Fig. 2). For the first three months after the initiation of new treatment in both arms, a treatment switch is only considered if the outcome is rated as"poor."After this period, a treatment switch is considered if the outcome is rated as"fair"or"poor."

### Outcomes

#### Primary endpoint

Reduction in number of patients treated with biologicals in the EXACT@home intervention arm compared to the control arm after 6 months follow-up.

#### Secondary endpoints

- Reduction in number of patients treated with biologicals in the EXACT@home intervention arm compared to the control arm after 12 months follow-up.

The difference in the following variables between the intervention—and control arm during follow-up is assessed:

- Asthma control: Asthma control is determined using the Asthma Control Questionnaire (ACQ) [49].
- Quality of life: Disease-specific quality of life is assessed using the mini Asthma Quality of Life Questionnaire (AQLQ) [50, 51].
- Exacerbation frequency: the number of exacerbations requiring prednisolone with or without antibiotics during the study period is documented.

### Data management

All personal data in this study will be handled in compliance with the EU General Data Protection Regulation (GDPR) and the Dutch Implementation Act of the General Data Protection Regulation (In Dutch: Uitvoeringswet AVG, UAVG). Patient identifiers will be pseudonymized in all databases and applications to ensure confidentiality.

### Sample size

In the absence of comparable studies, we aim to assess the reduction of biologicals in the intervention arm compared to the control arm. We expect approximately 75% of patients to remain on the initial biological at the end of the study [10, 52] and ≤ 45% of patients in the intervention arm ultimately requiring a biological [21]. With 80% power, an alpha of 0.01 (Bonferroni correction for 5 primary and secondary parameters), a superiority margin of 0, a sampling ratio of 1, and expected proportions of 45% in the intervention arm and 75% in the control arm, a two-tailed superiority test comparing two proportions (taking into account a total dropout of 20%) results in 69 patients in each arm.

### Randomisation

Patients will be randomly assigned to either the intervention or control arm in a 1:1 ratio using variable block randomization via Castor EDC, performed by the coordinating investigator. The block sizes will be 2, 4, and 6. Blinding between the two arms is not possible due to the study design.

### Statistical analysis

This study will use an intention-to-treat analysis (primary analysis), incorporating data from all included patients, including those who drop out, change their initial treatment, or have protocol non-compliance. Additionally, a per-protocol analysis will be performed. To determine if there is a significant reduction in prescription of biologicals in the intervention arm compared to the control arm at 6 and 12 months, a Chi-squared test will be applied. A repeated measurements analysis approach will be applied to assess the differences between the intervention and control arm and/or changes over time for all other secondary variables.

### Safety

Serious adverse (device) events (SA(D)Es) are systematically monitored and reported in accordance with regulatory requirements and timelines. Although this is a low-risk, open-label study, a Data and Safety Monitoring Board (DSMB) is established to provide safety monitoring. The DSMB will conduct periodic reviews of safety (SA(D)Es), feasibility, and study futility every six months. In addition, since the BF-digihaler-DS has not yet received authorization from the European Medicines Agency (EMA), safety assessments will be carried out throughout the study. However, the BF-digihaler is identical to the DuoResp Spiromax 160 µg/4.5 µg by Teva®, aside from the addition of the digital system (DS), is already available on the market for use in standard asthma care. Therefore, no significant risks are anticipated.

## Discussion

Although severe asthma represents a minority of the asthma population, it accounts for a significant healthcare and disease burden. Its heterogeneous nature requires a personalised approach, breaking down the disease into treatable traits unique to each individual. In severe cases, these traits are often hidden, making the disease seem refractory. A multi-dimensional assessment is therefore crucial, categorising traits into pulmonary, extrapulmonary and behavioural/lifestyle domains, followed by tailored treatments based on the identified traits [4, 23, 53]. Effective identification and treatment of these traits can potentially reduce the need for costly asthma biologicals.

While existing literature provides limited evidence on the impact of a treatable trait-based multidimensional assessment in difficult-to-treat or severe asthma examining different clinical outcome measures, there’s a general consensus on its potential positive effects. In addition, the role of digital home monitoring in identifying and managing treatable traits, as well as the potential reduction in biological prescriptions resulting from systematic assessment remains underexplored.

In conclusion, we identified a lack of high-quality data on the effect of a systematic assessment of treatable traits specifically concerning digital home monitoring and its influence on biological prescription. With this open label, randomized controlled trial we aim to provide evidence on the impact of such a systematic assessment on biological prescription, asthma control, quality of life and exacerbation frequency.

## Dissemination policy

The results will be made publicly available through presentations at national and international conferences, as well as through publication in scientific journals. The funding parties will have no involvement in the disclosure of the research findings.

## Supplementary Information

Additional file 1

Additional file 2

Additional file 3

Additional file 4

Additional file 5

## Data Availability

All data relevant to the study are included in the article or uploaded as supplementary information. The datasets used and/or analyzed during the current study are available from the corresponding author on reasonable request.

## Abbreviations

ICS: Inhaled corticosteroids
(m)OCS: (Maintenance) oral corticosteroids
PROM: Patient reported outcome measure
ACT and ACQ: Asthma control test and questionnaire
AQLQ: Asthma quality of life questionnaire
HCU: Health care utilization
FEV1: Forced expiratory volume in 1 s
TRA: Therapy resistant asthma
GERD: Gastroesophageal reflux disease
MDC: Multi-disciplinary consultation
PDHE: Personal digital healthcare environment
FeNO: Fractional exhaled Nitric Oxide
DPI: Dry powder inhaler
NCSI: Nijmegen Screening Clinical Instrument
6MWT: 6-Minute Walk Test
SPPB: Short physical performance battery
LABA: Long-acting beta-agonist
GINA: Global Initiative for Asthma
GETE: Global evaluation of treatment effectiveness
GDPR: General Data Protection Regulation
SA(D)E: Serious adverse (device) events
DSMB: Data safety monitoring board
mMRC: (Modified) Medical Research Council dyspnea scale
