# Supplementary figures and images for: The effect of a systematic multi-dimensional assessment in severe uncontrolled asthma: a literature review and protocol for an investigator-initiated, open-label, randomized-controlled trial (EXACT@home study)
Source: BMC Pulm Med. 2025 May 17;25:240. doi: 10.1186/s12890-025-03646-5 (PMC12085824; doi:10.1186/s12890-025-03646-5)

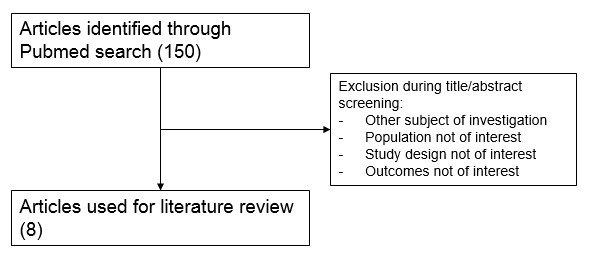

Supplement: Supplementary file 1 — Additional file 1 [file 12890_2025_3646_MOESM1_ESM.jpg]

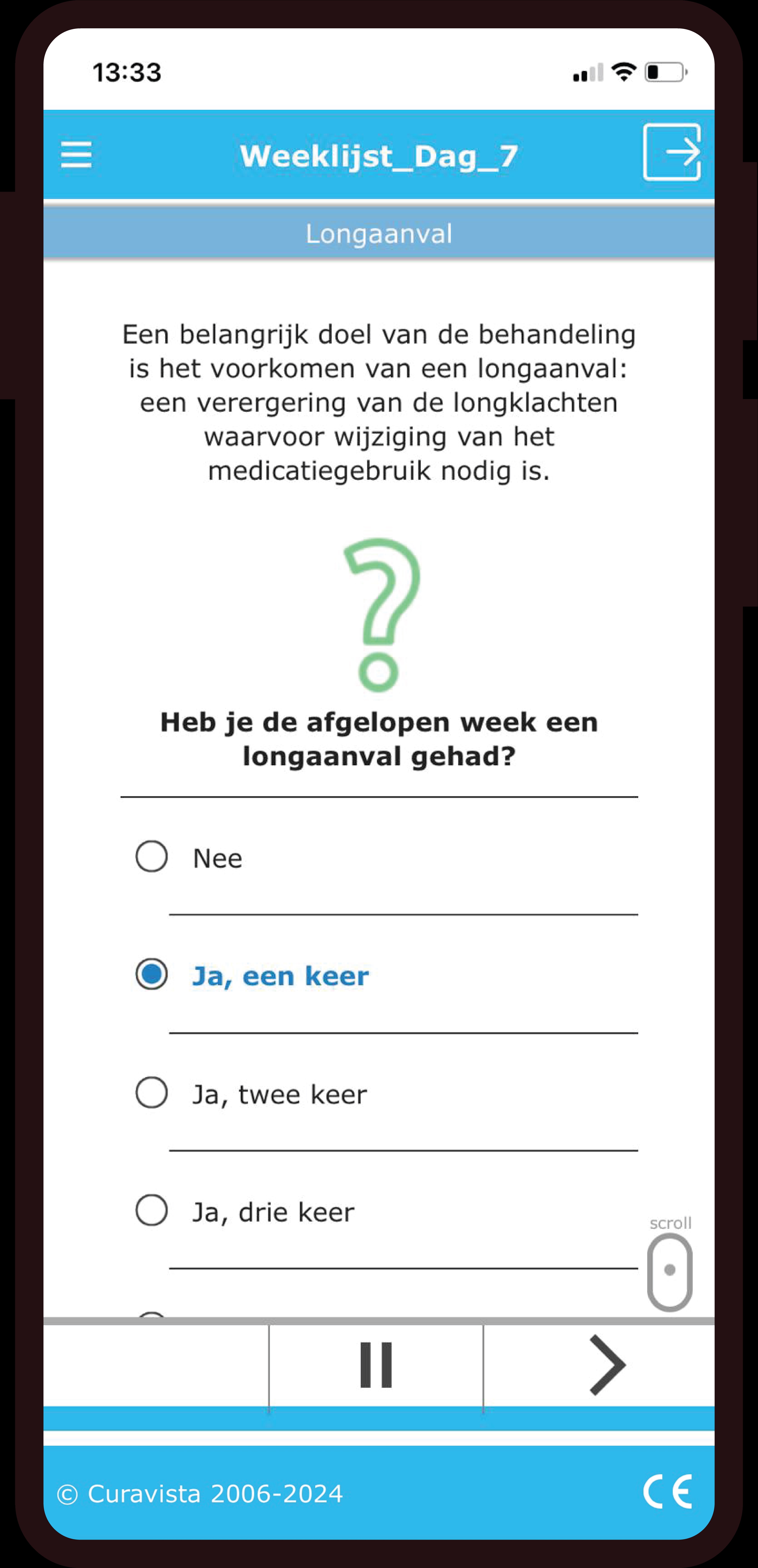

Supplement: Supplementary file 3 — Additional file 3 [file 12890_2025_3646_MOESM3_ESM.zip › Suppl. 3 - Figure 2a.jpg]

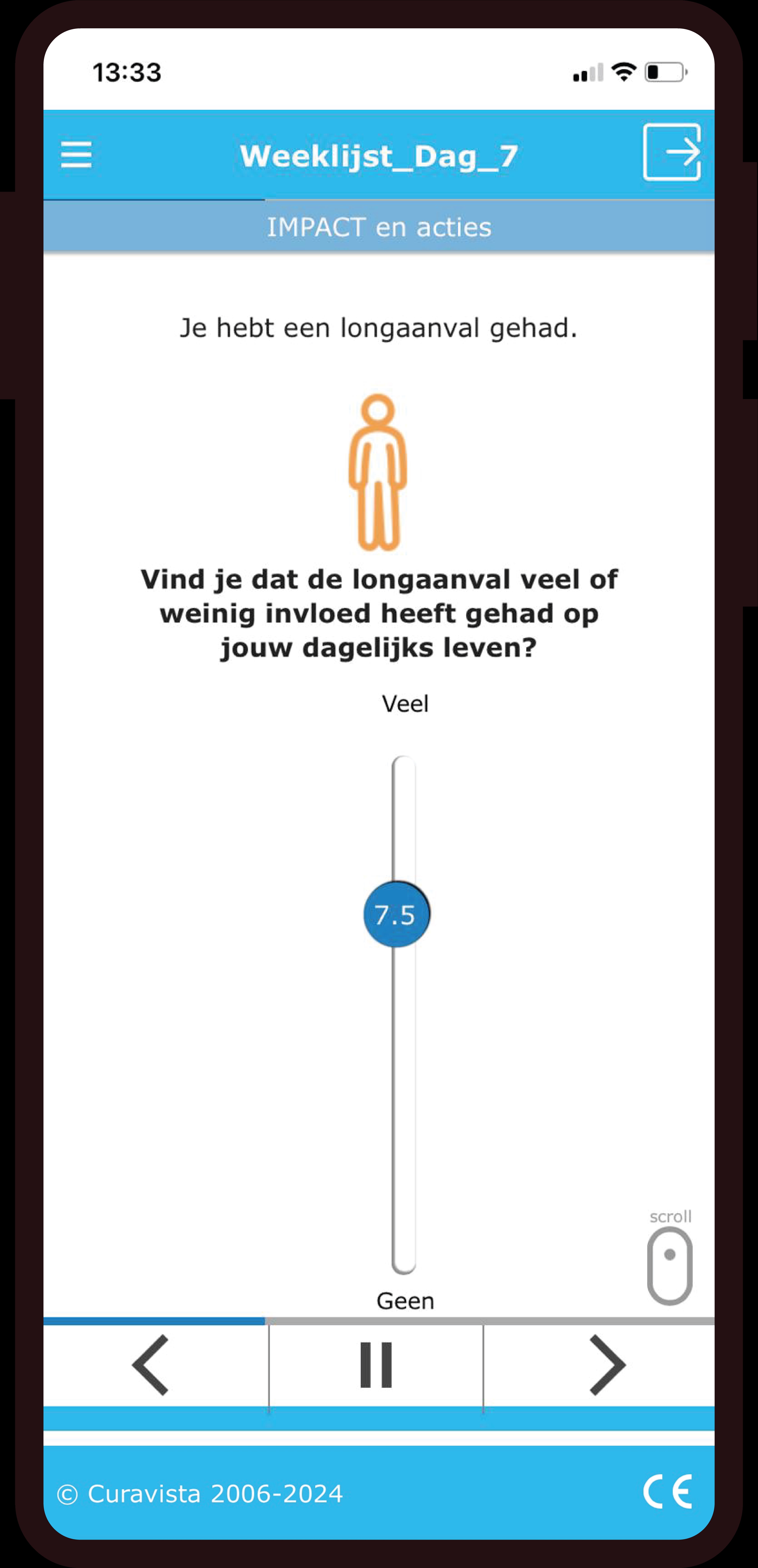

Supplement: Supplementary file 3 — Additional file 3 [file 12890_2025_3646_MOESM3_ESM.zip › Suppl. 3 - Figure 2b.jpg]

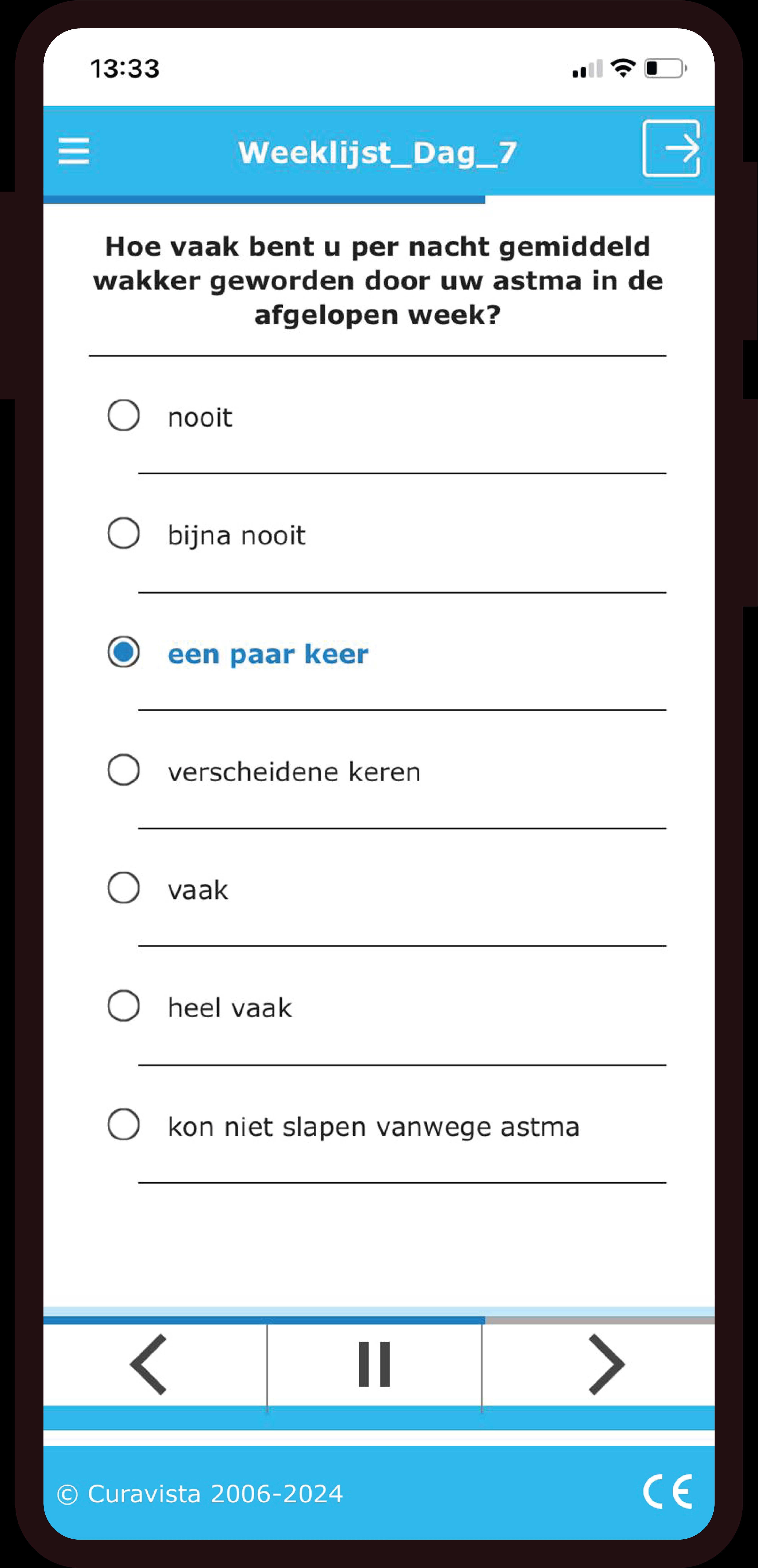

Supplement: Supplementary file 3 — Additional file 3 [file 12890_2025_3646_MOESM3_ESM.zip › Suppl. 3 - Figure 2c.jpg]

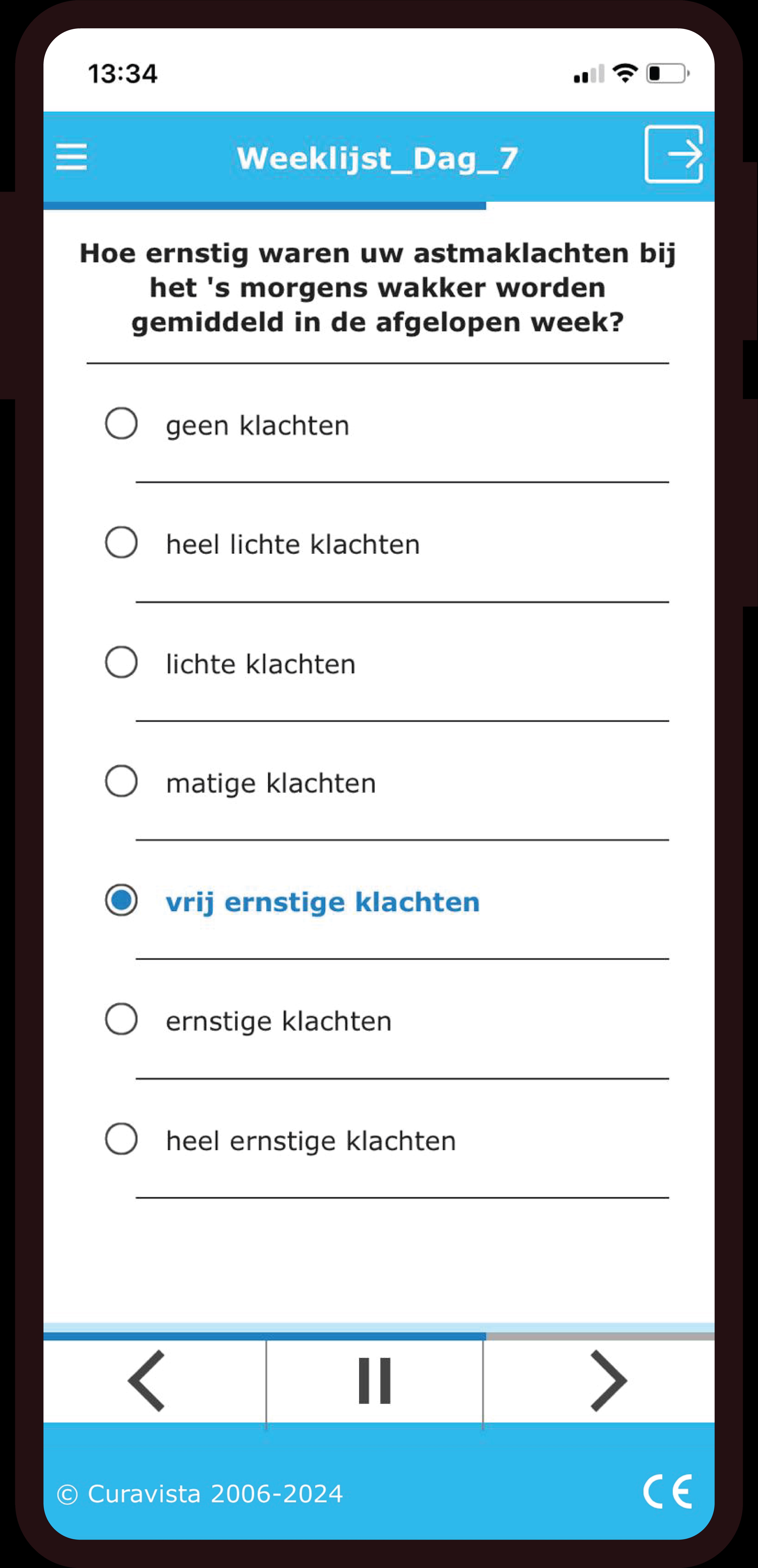

Supplement: Supplementary file 3 — Additional file 3 [file 12890_2025_3646_MOESM3_ESM.zip › Suppl. 3 - Figure 2d.jpg]

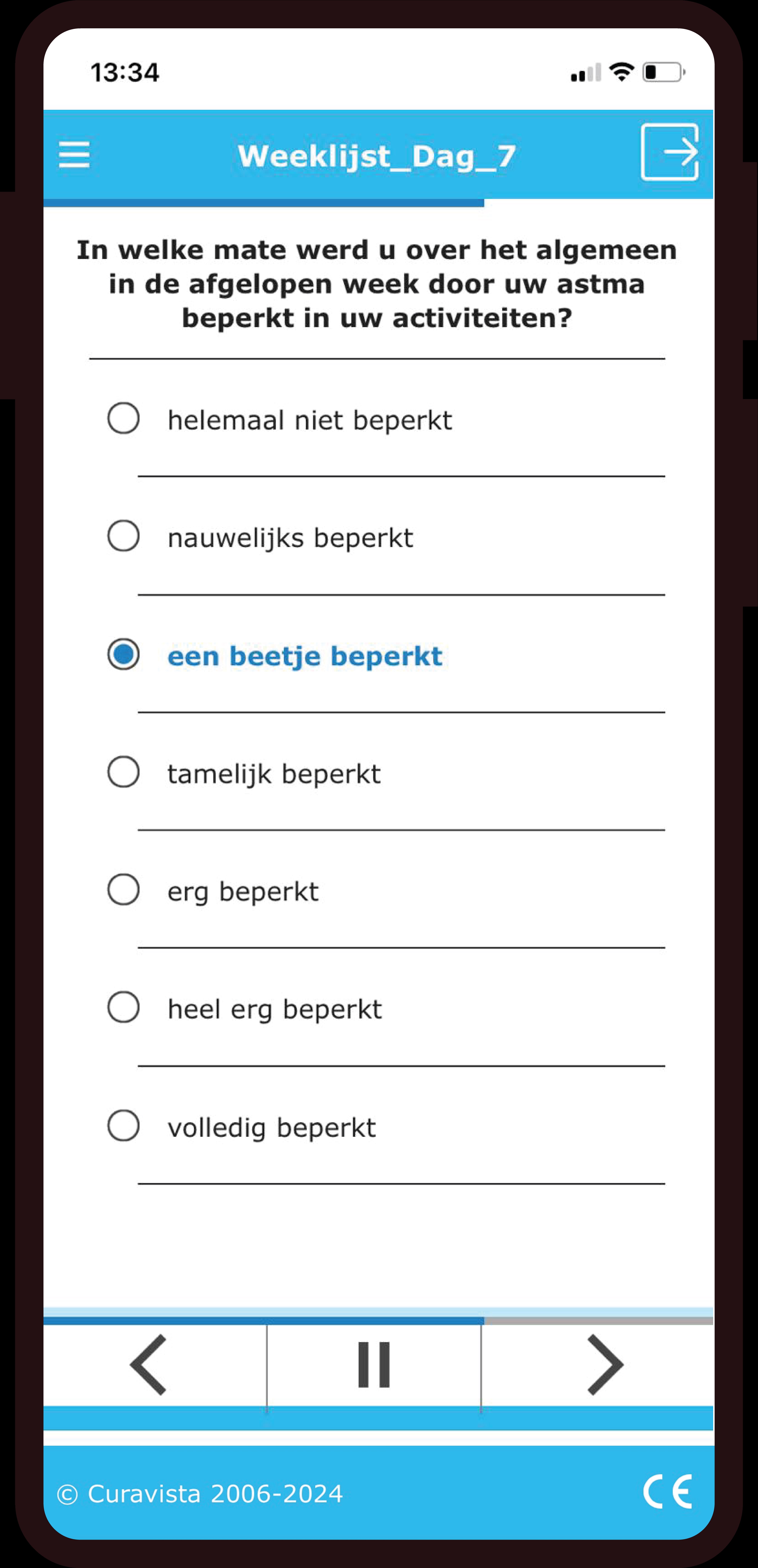

Supplement: Supplementary file 3 — Additional file 3 [file 12890_2025_3646_MOESM3_ESM.zip › Suppl. 3 - Figure 2e.jpg]

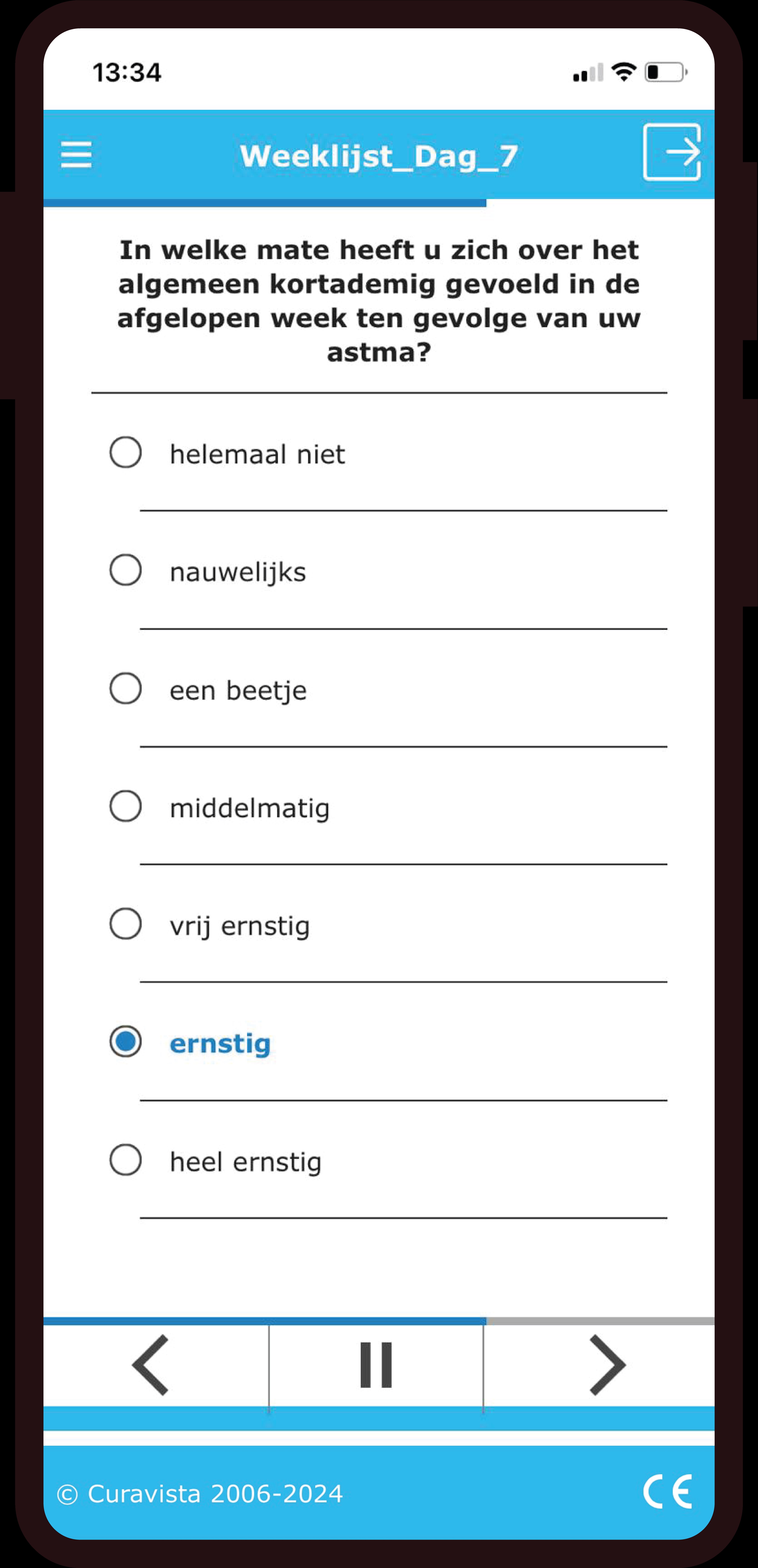

Supplement: Supplementary file 3 — Additional file 3 [file 12890_2025_3646_MOESM3_ESM.zip › Suppl. 3 - Figure 2f.jpg]

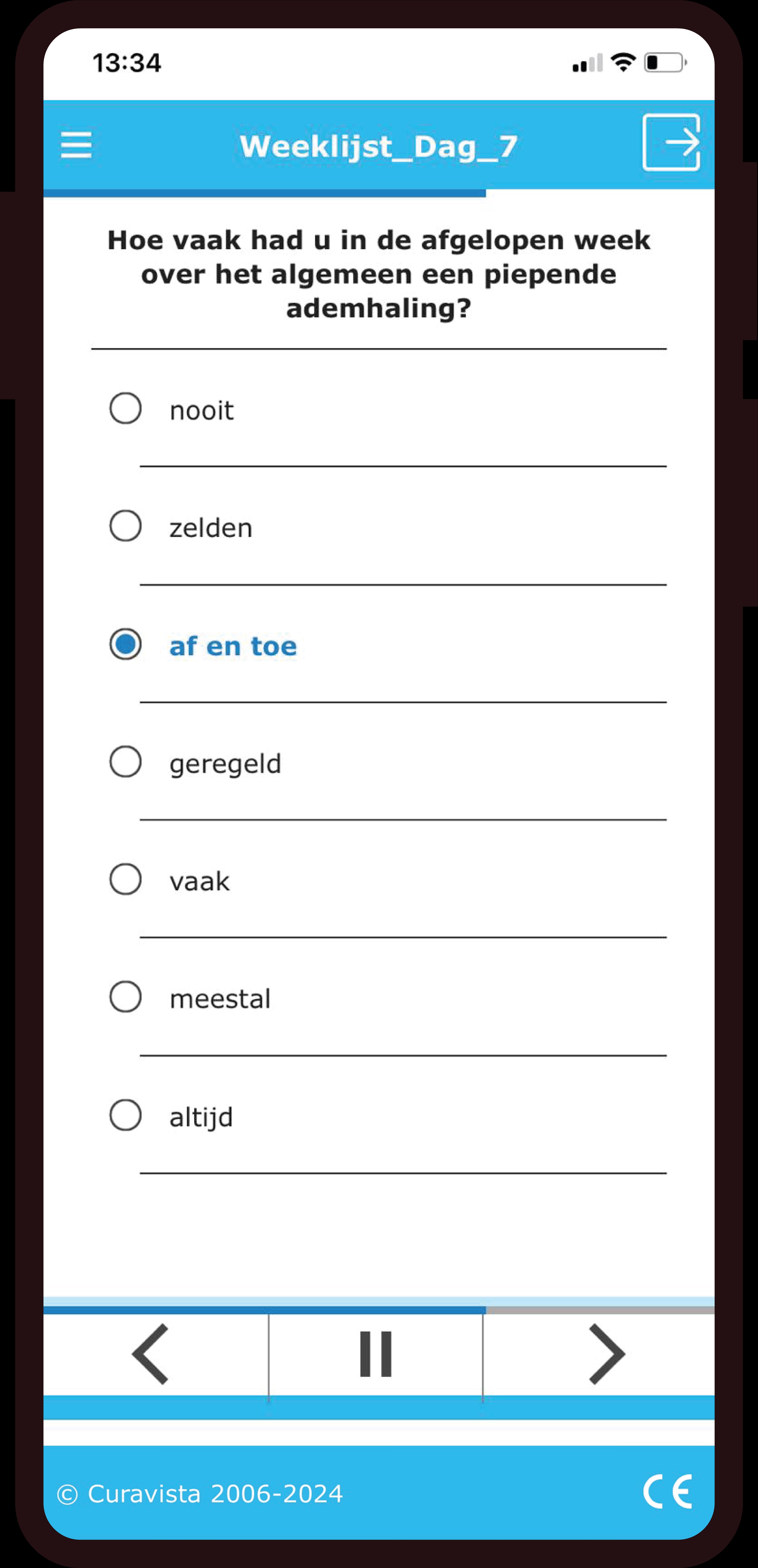

Supplement: Supplementary file 3 — Additional file 3 [file 12890_2025_3646_MOESM3_ESM.zip › Suppl. 3 - Figure 2g.jpg]

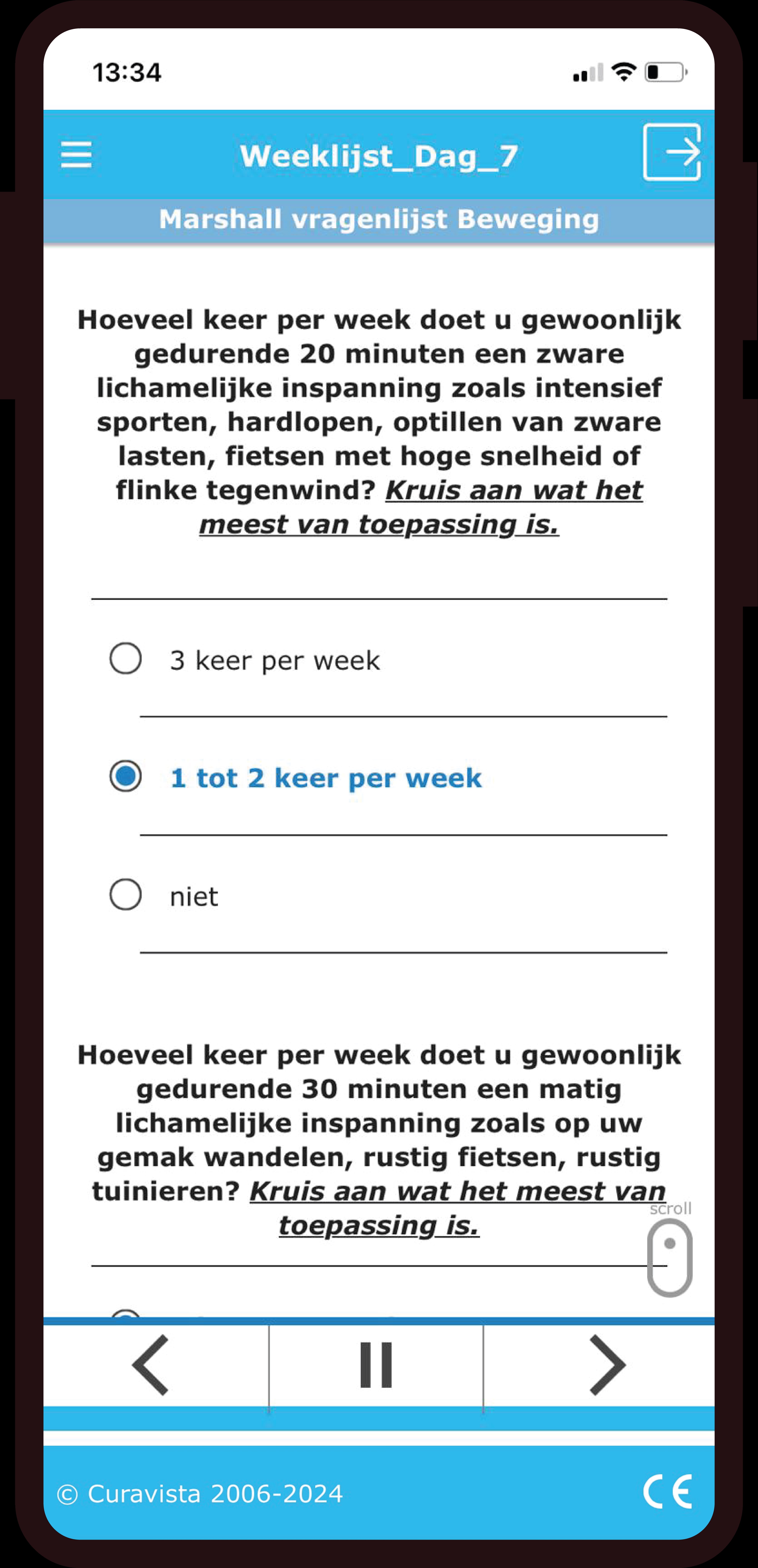

Supplement: Supplementary file 3 — Additional file 3 [file 12890_2025_3646_MOESM3_ESM.zip › Suppl. 3 - Figure 2h.jpg]

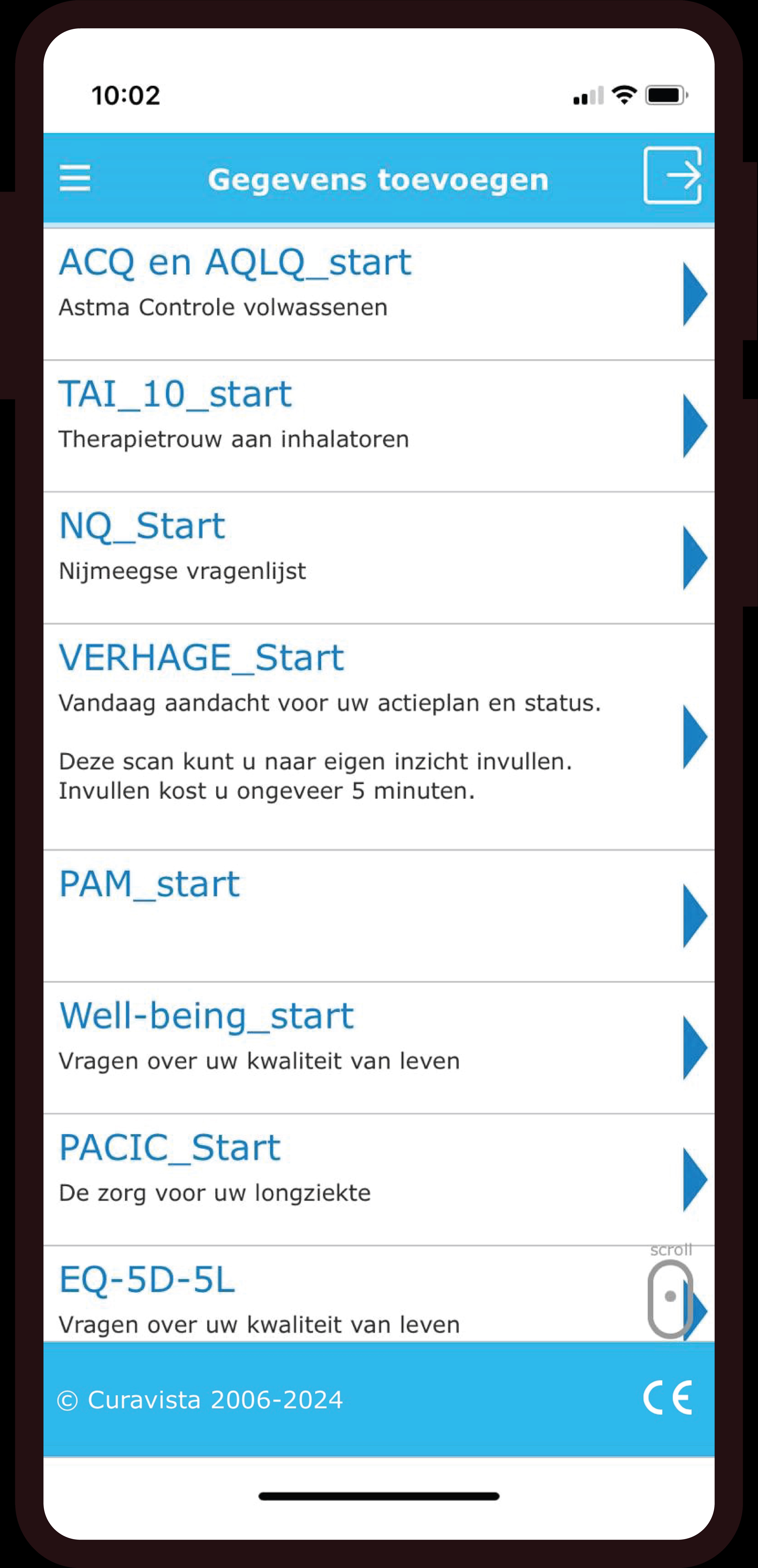

Supplement: Supplementary file 3 — Additional file 3 [file 12890_2025_3646_MOESM3_ESM.zip › Suppl. 3 - Figure 3.jpg]

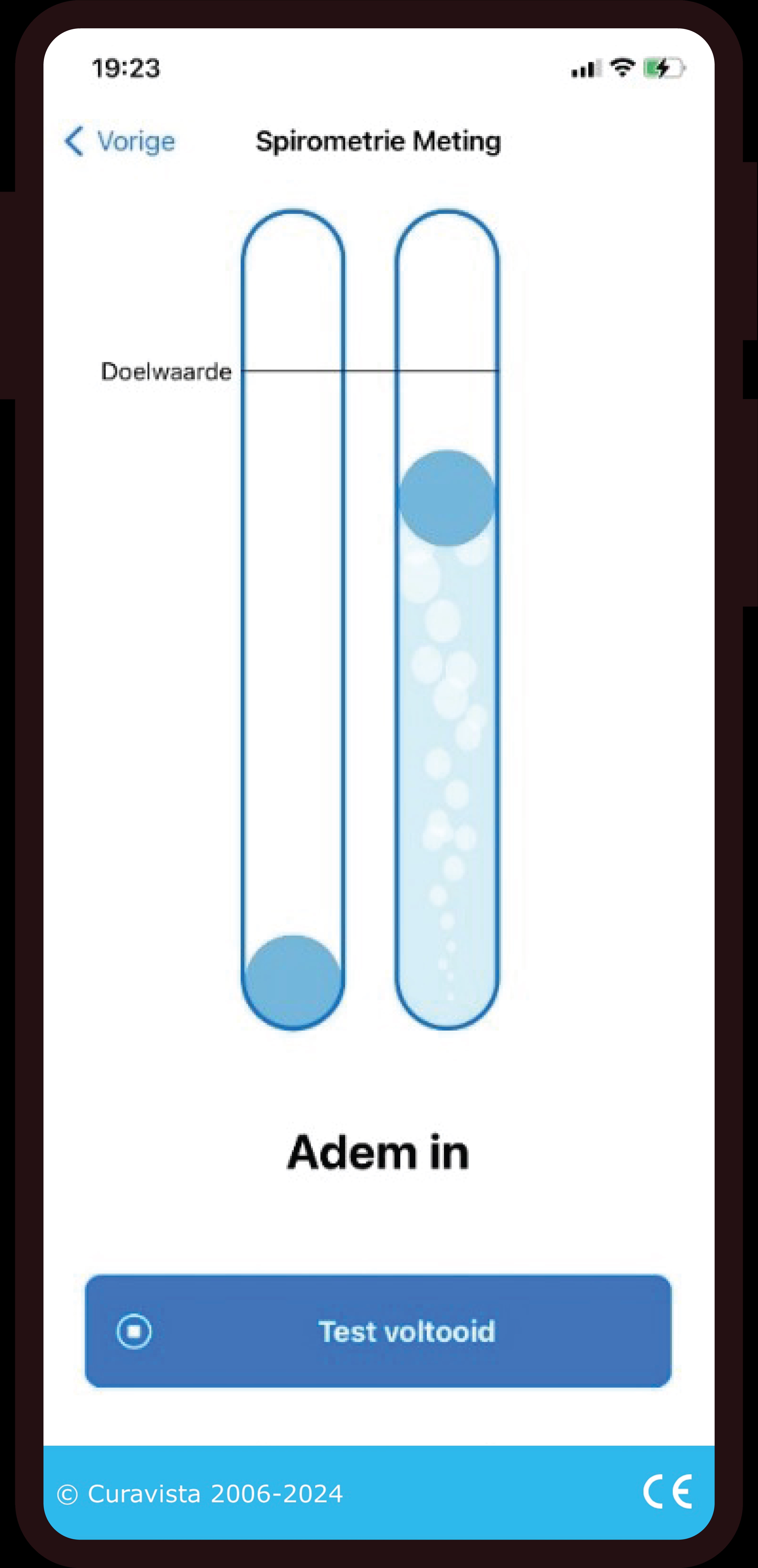

Supplement: Supplementary file 3 — Additional file 3 [file 12890_2025_3646_MOESM3_ESM.zip › Suppl. 3 - Figure 4a.jpg]

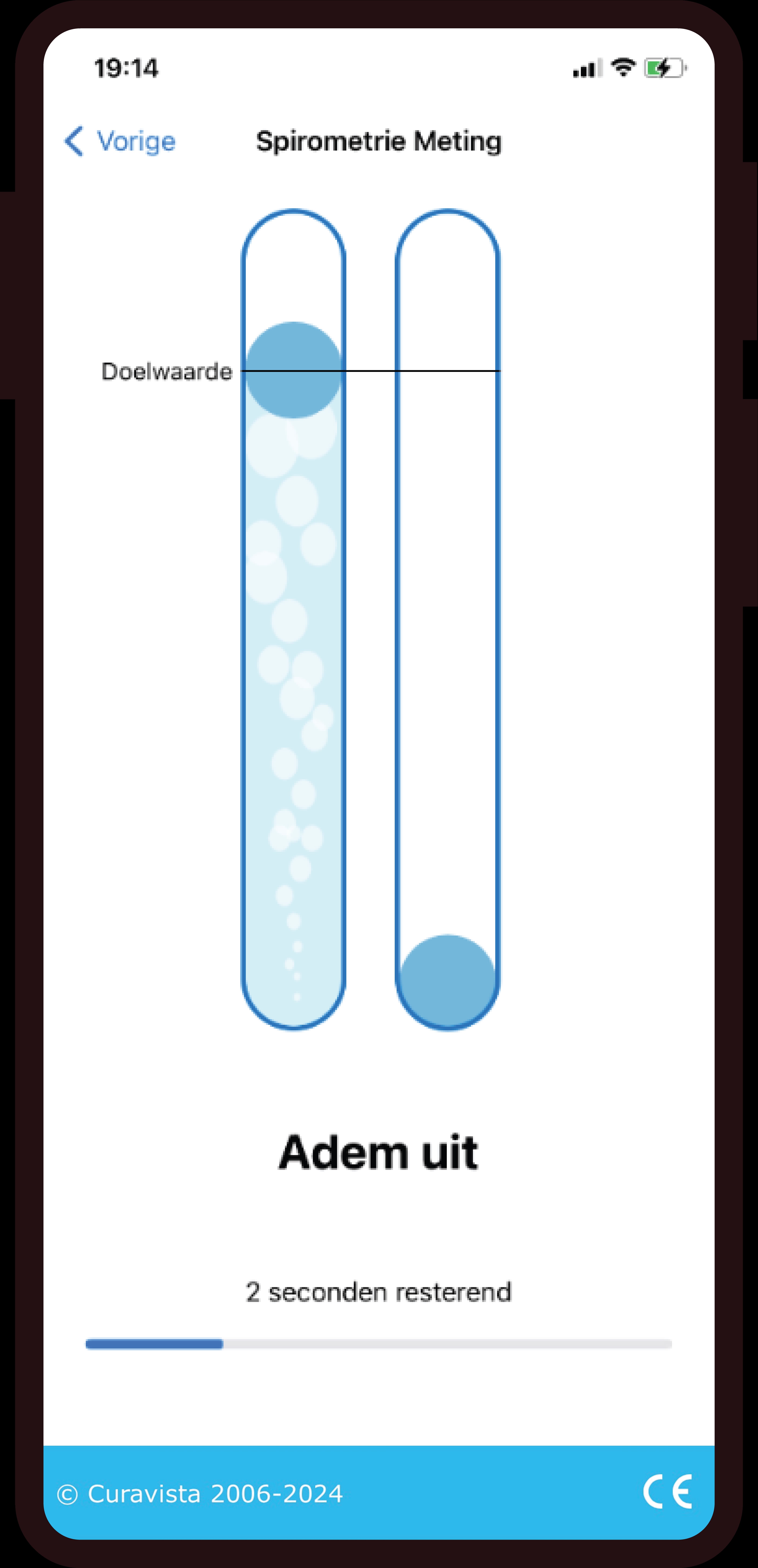

Supplement: Supplementary file 3 — Additional file 3 [file 12890_2025_3646_MOESM3_ESM.zip › Suppl. 3 - Figure 4b.jpg]

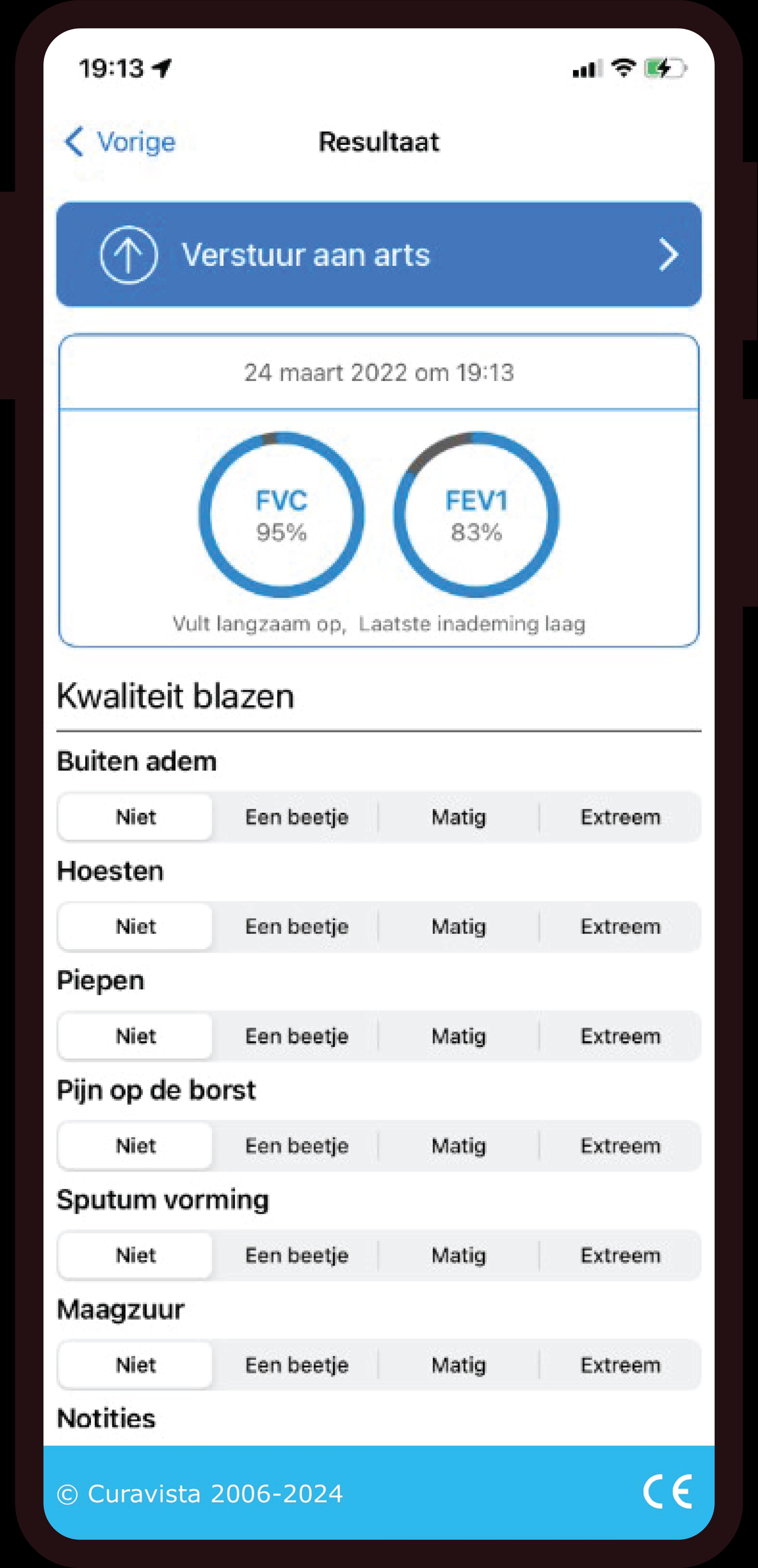

Supplement: Supplementary file 3 — Additional file 3 [file 12890_2025_3646_MOESM3_ESM.zip › Suppl. 3 - Figure 4c.jpg]

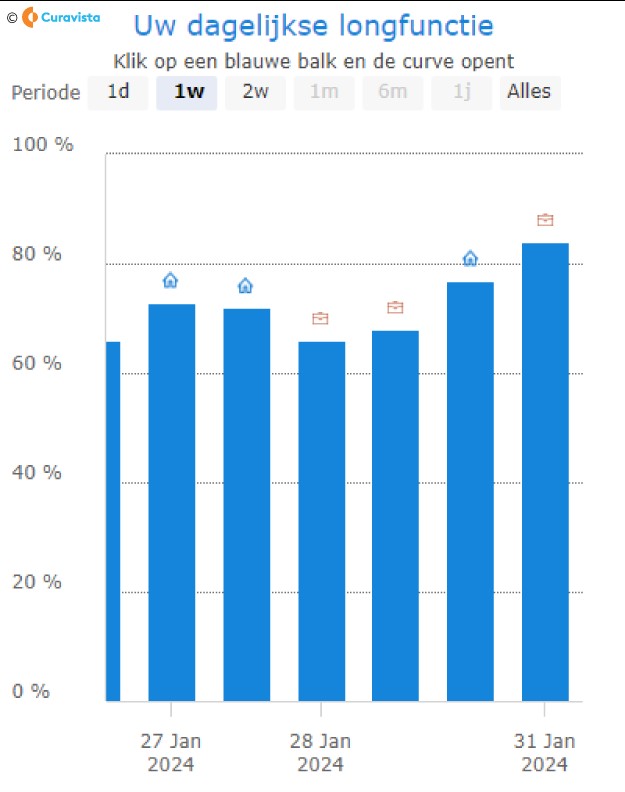

Supplement: Supplementary file 3 — Additional file 3 [file 12890_2025_3646_MOESM3_ESM.zip › Suppl. 3 - Figure 5.jpg]

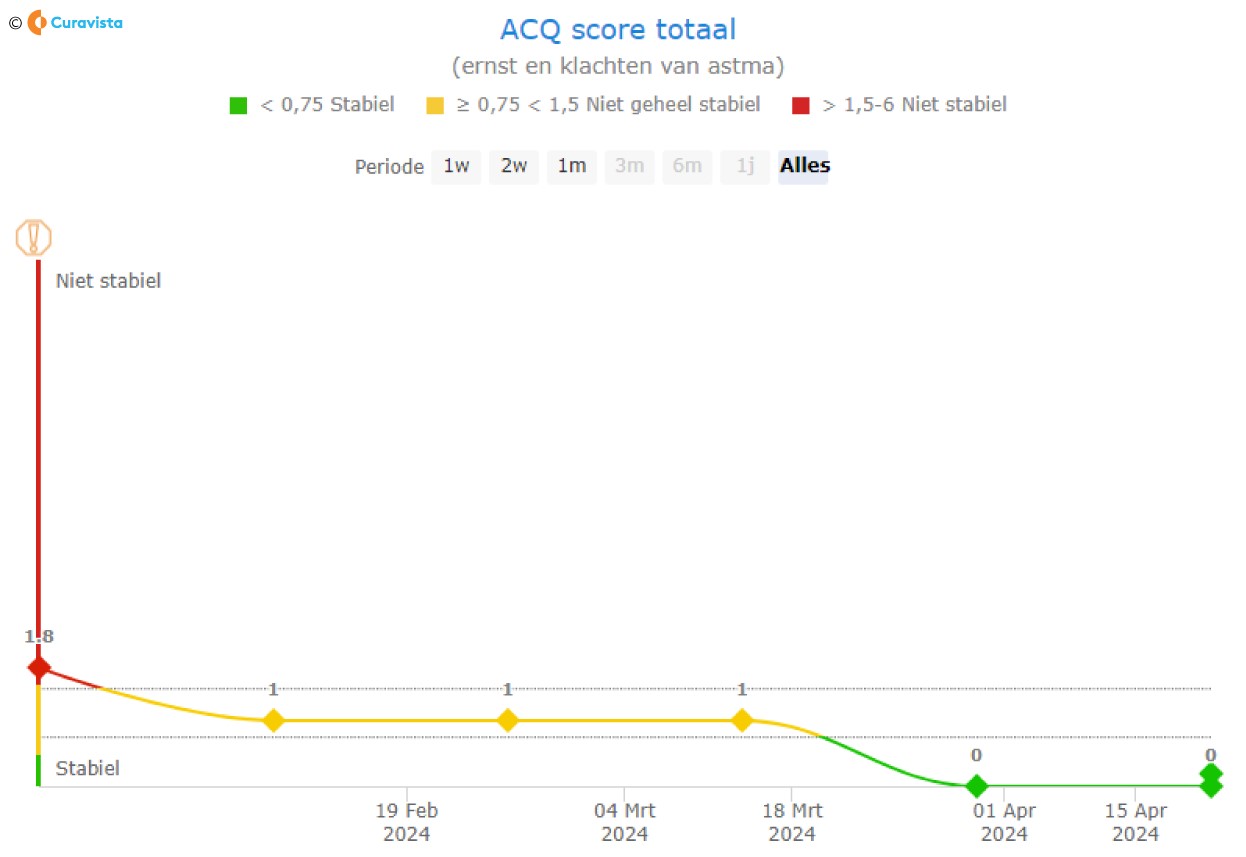

Supplement: Supplementary file 3 — Additional file 3 [file 12890_2025_3646_MOESM3_ESM.zip › Suppl. 3 - Figure 6.jpg]

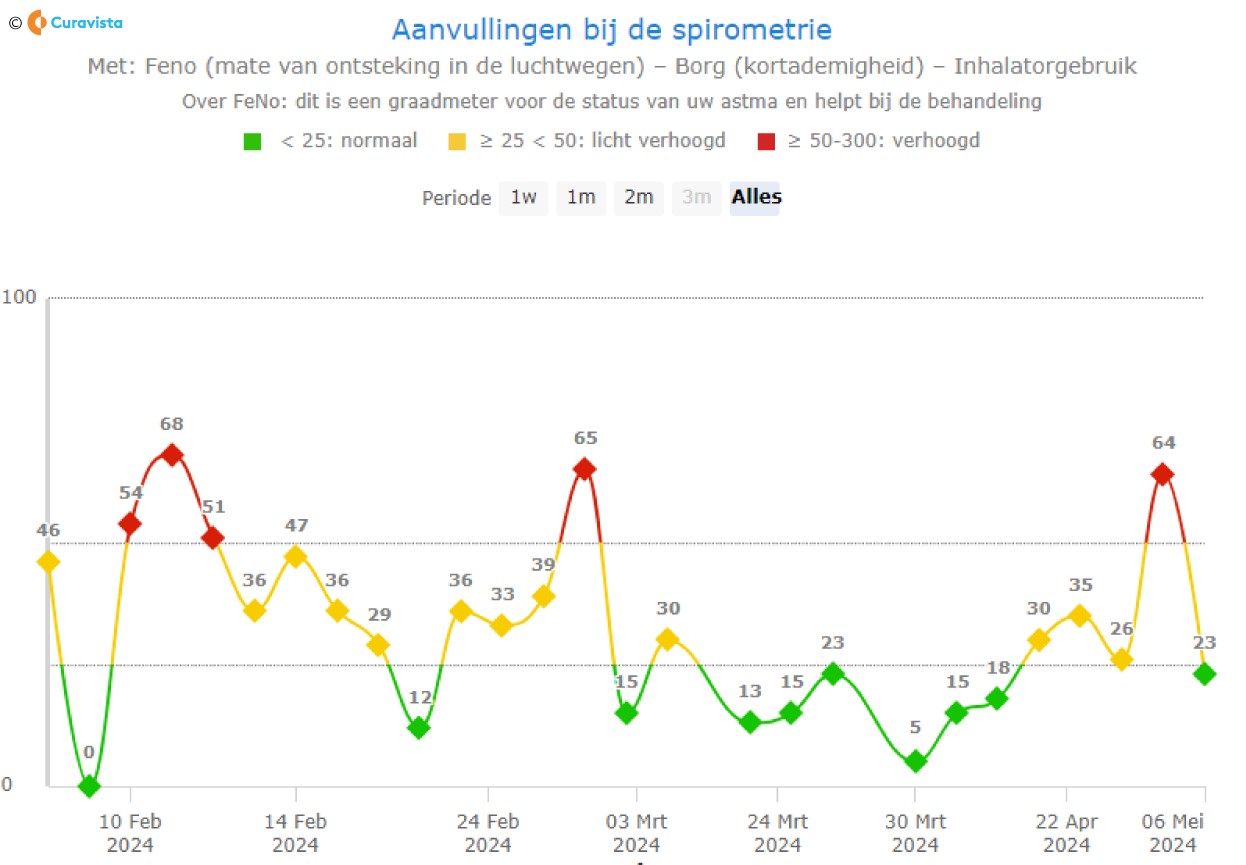

Supplement: Supplementary file 3 — Additional file 3 [file 12890_2025_3646_MOESM3_ESM.zip › Suppl. 3 - Figure 8.jpg]

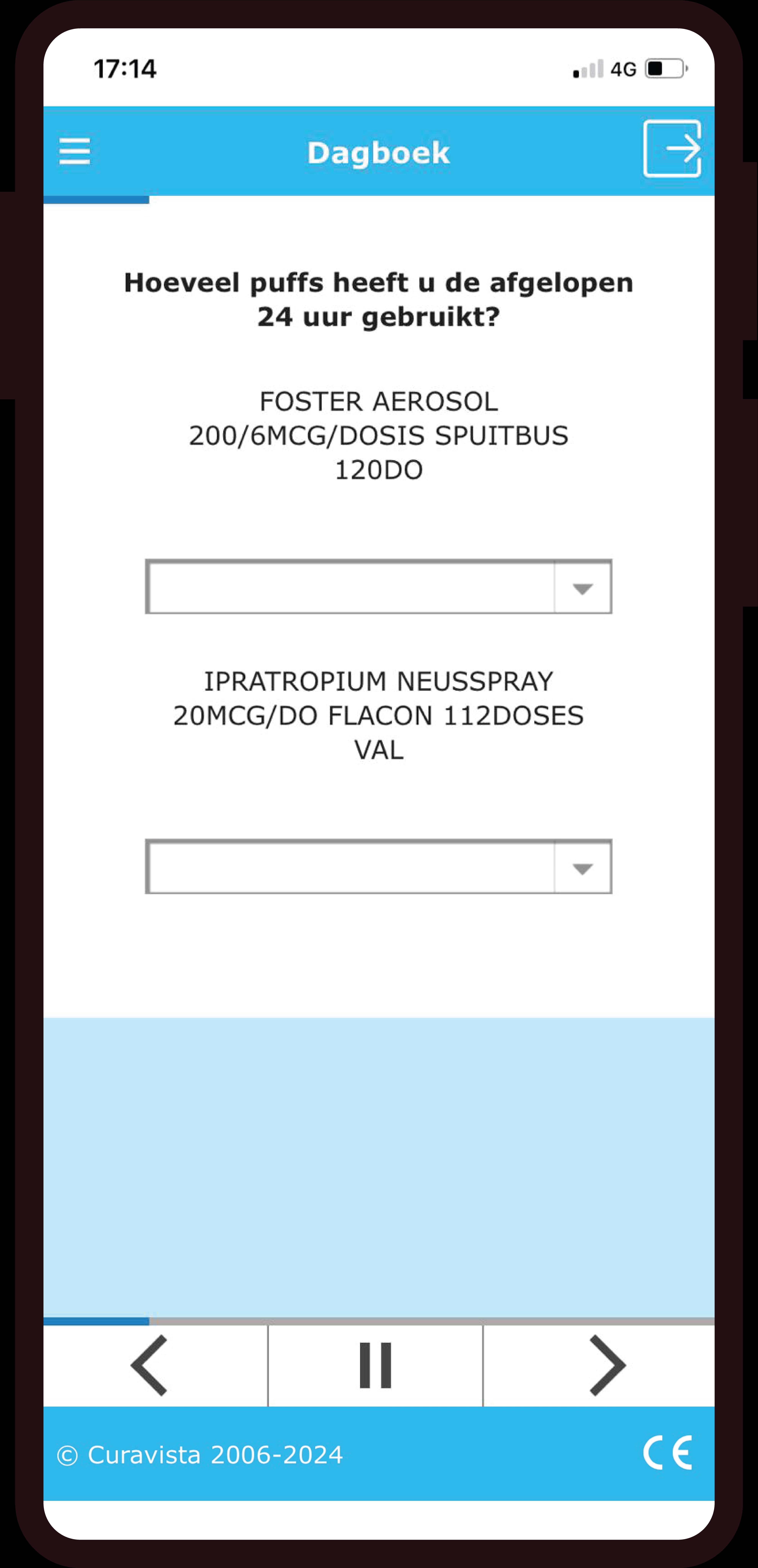

Supplement: Supplementary file 3 — Additional file 3 [file 12890_2025_3646_MOESM3_ESM.zip › Suppl. 3 Figure 1a.jpg]

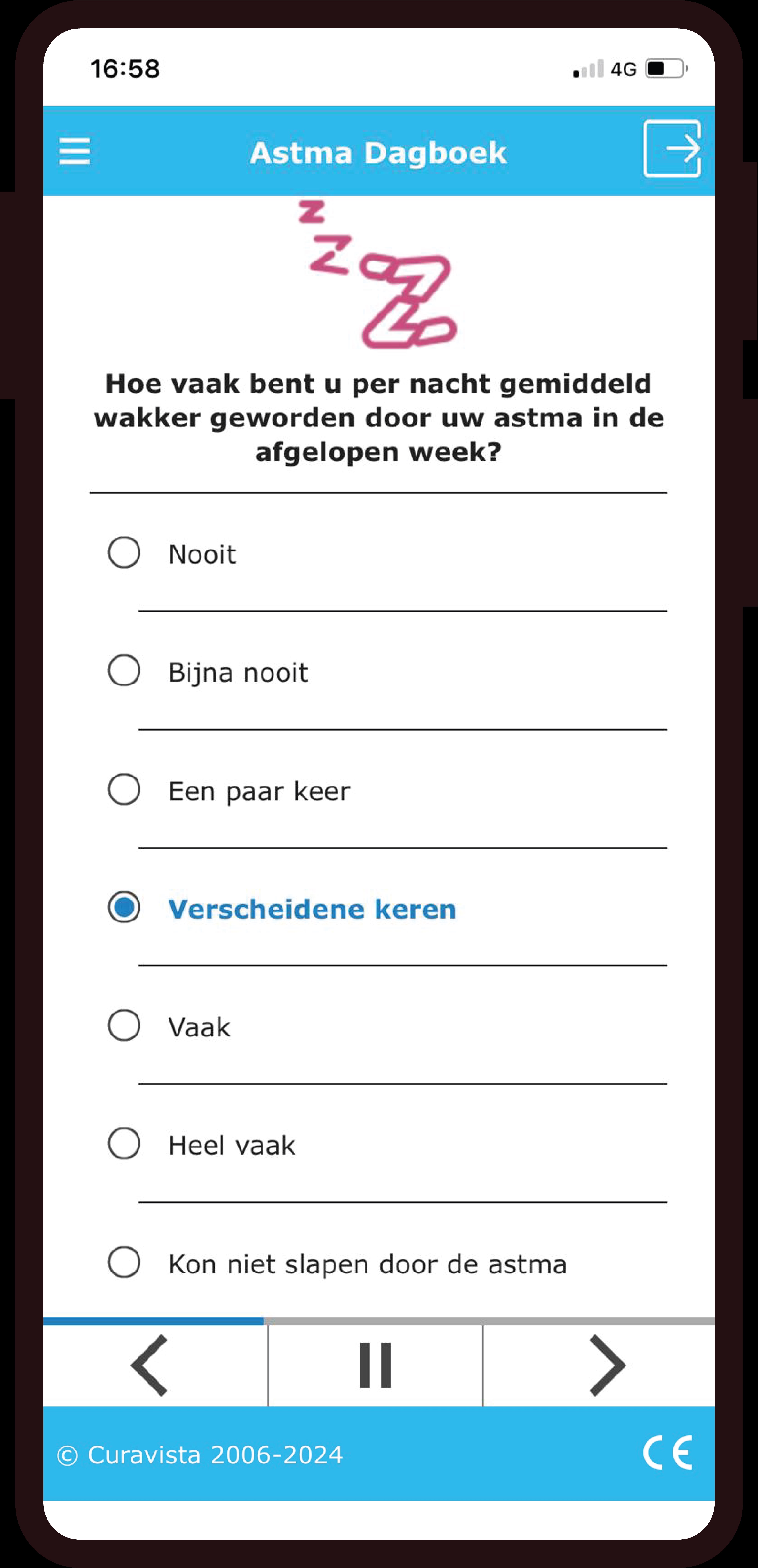

Supplement: Supplementary file 3 — Additional file 3 [file 12890_2025_3646_MOESM3_ESM.zip › Suppl. 3 Figure 1b.jpg]

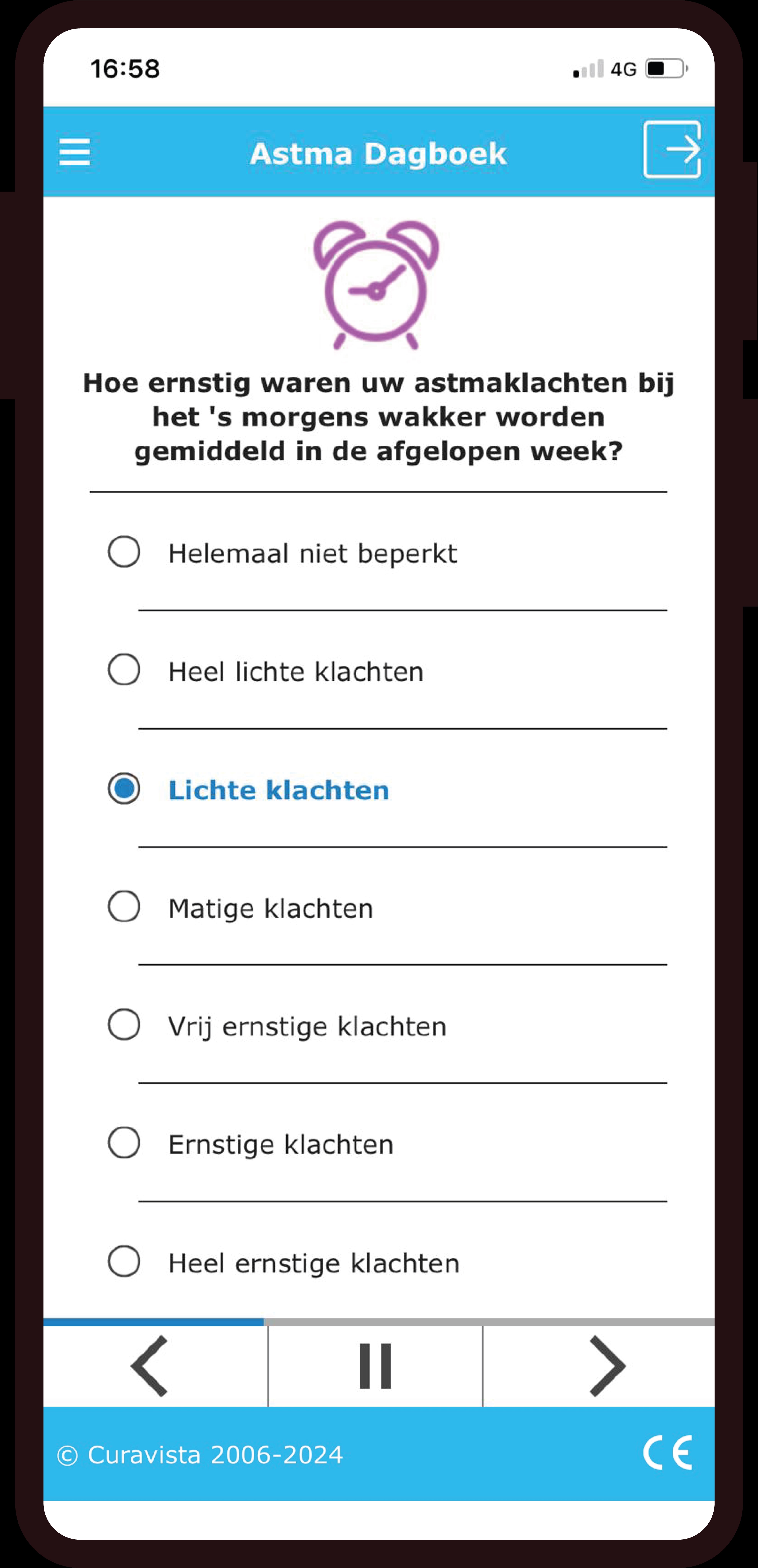

Supplement: Supplementary file 3 — Additional file 3 [file 12890_2025_3646_MOESM3_ESM.zip › Suppl. 3 Figure 1c.jpg]

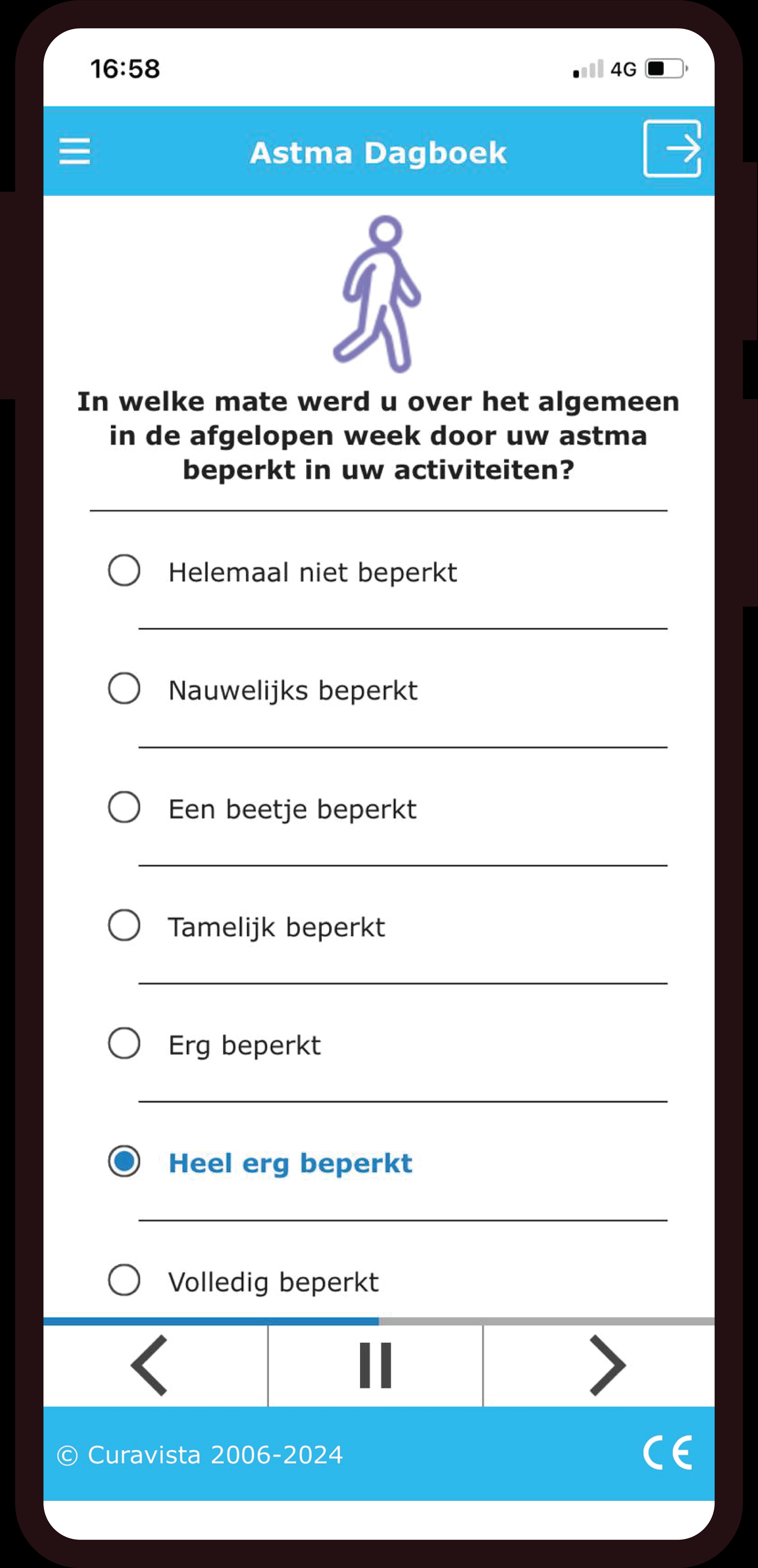

Supplement: Supplementary file 3 — Additional file 3 [file 12890_2025_3646_MOESM3_ESM.zip › Suppl. 3 Figure 1d.jpg]

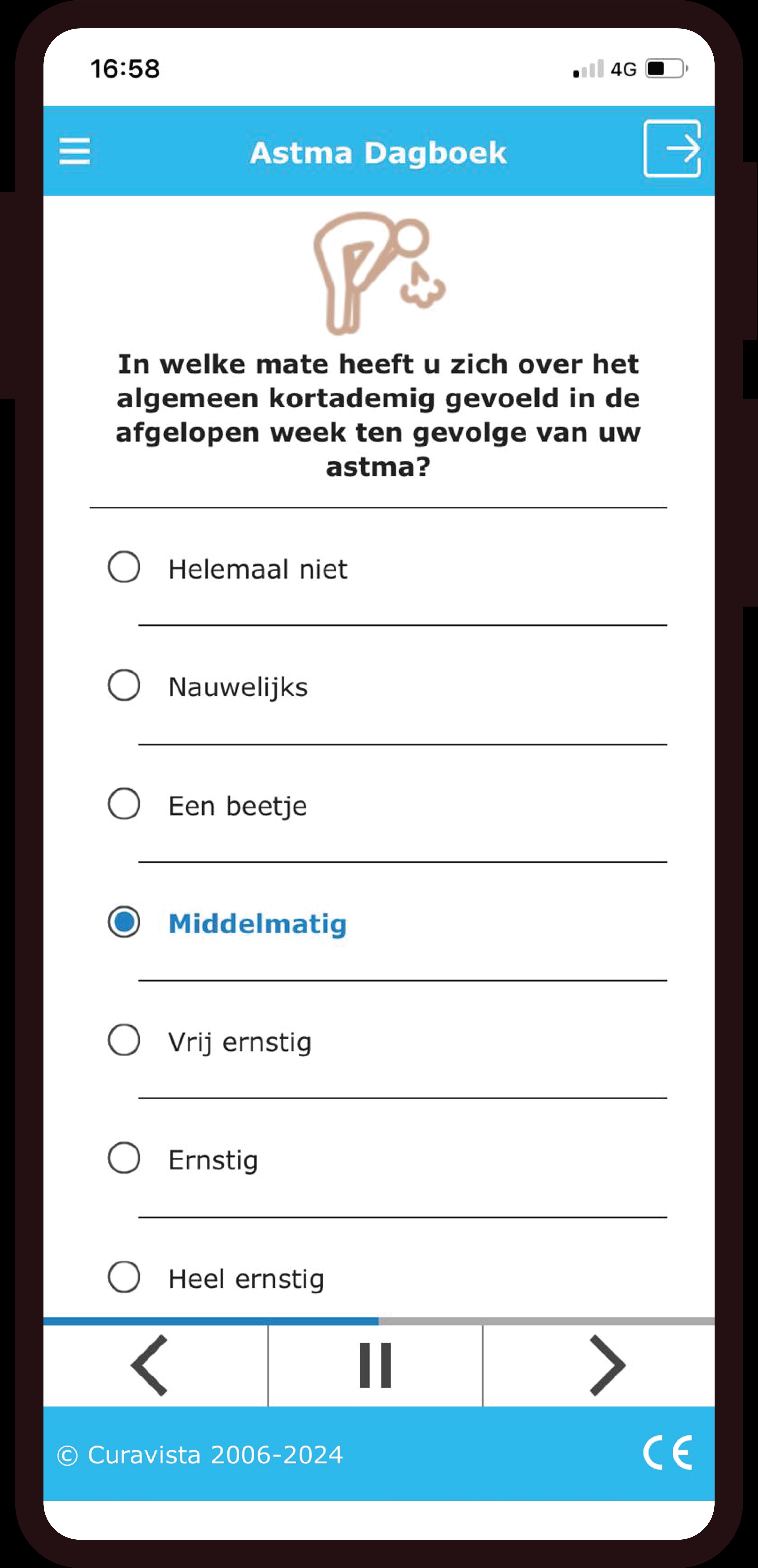

Supplement: Supplementary file 3 — Additional file 3 [file 12890_2025_3646_MOESM3_ESM.zip › Suppl. 3 Figure 1e.jpg]

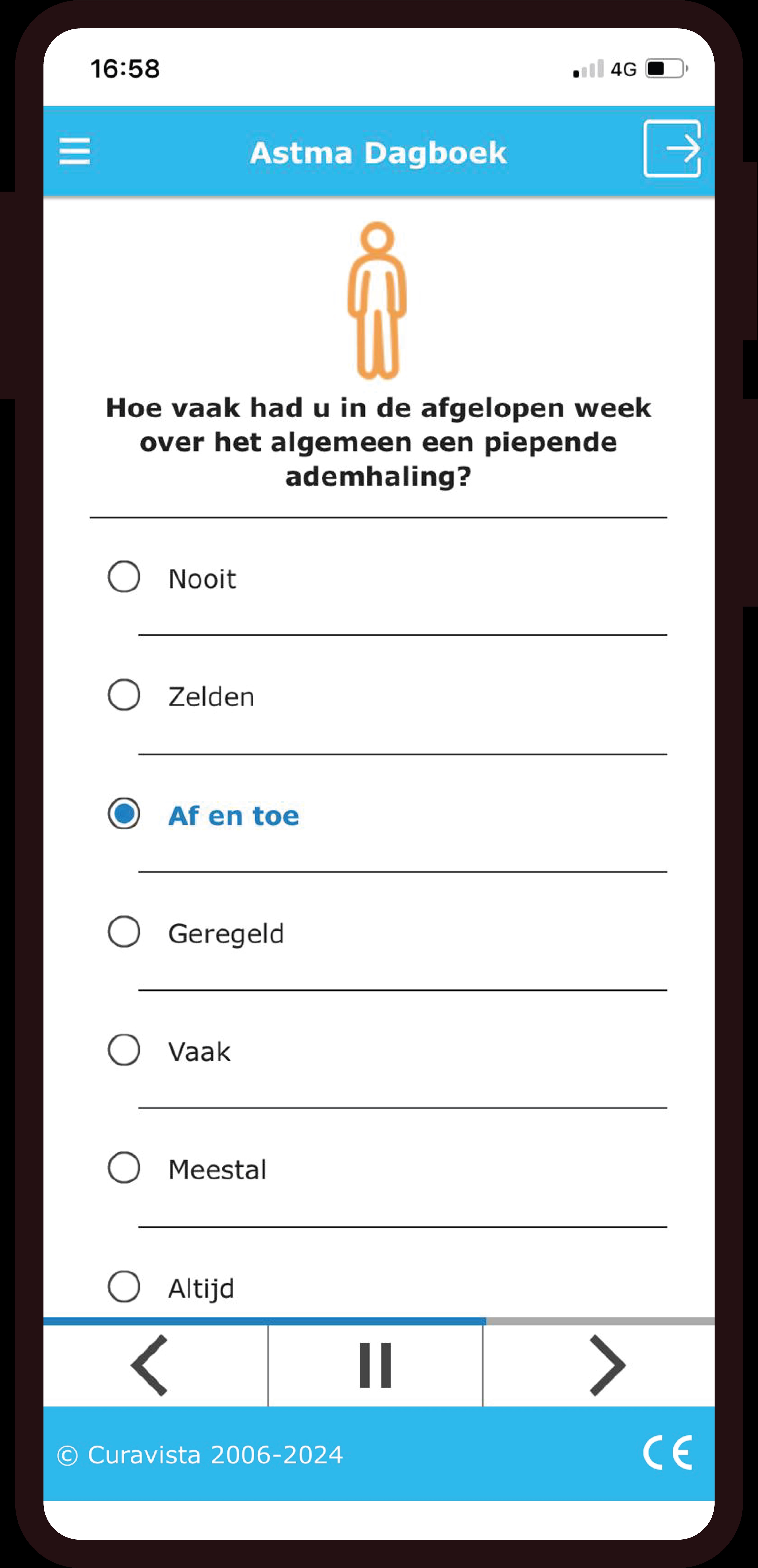

Supplement: Supplementary file 3 — Additional file 3 [file 12890_2025_3646_MOESM3_ESM.zip › Suppl. 3 Figure 1f.jpg]

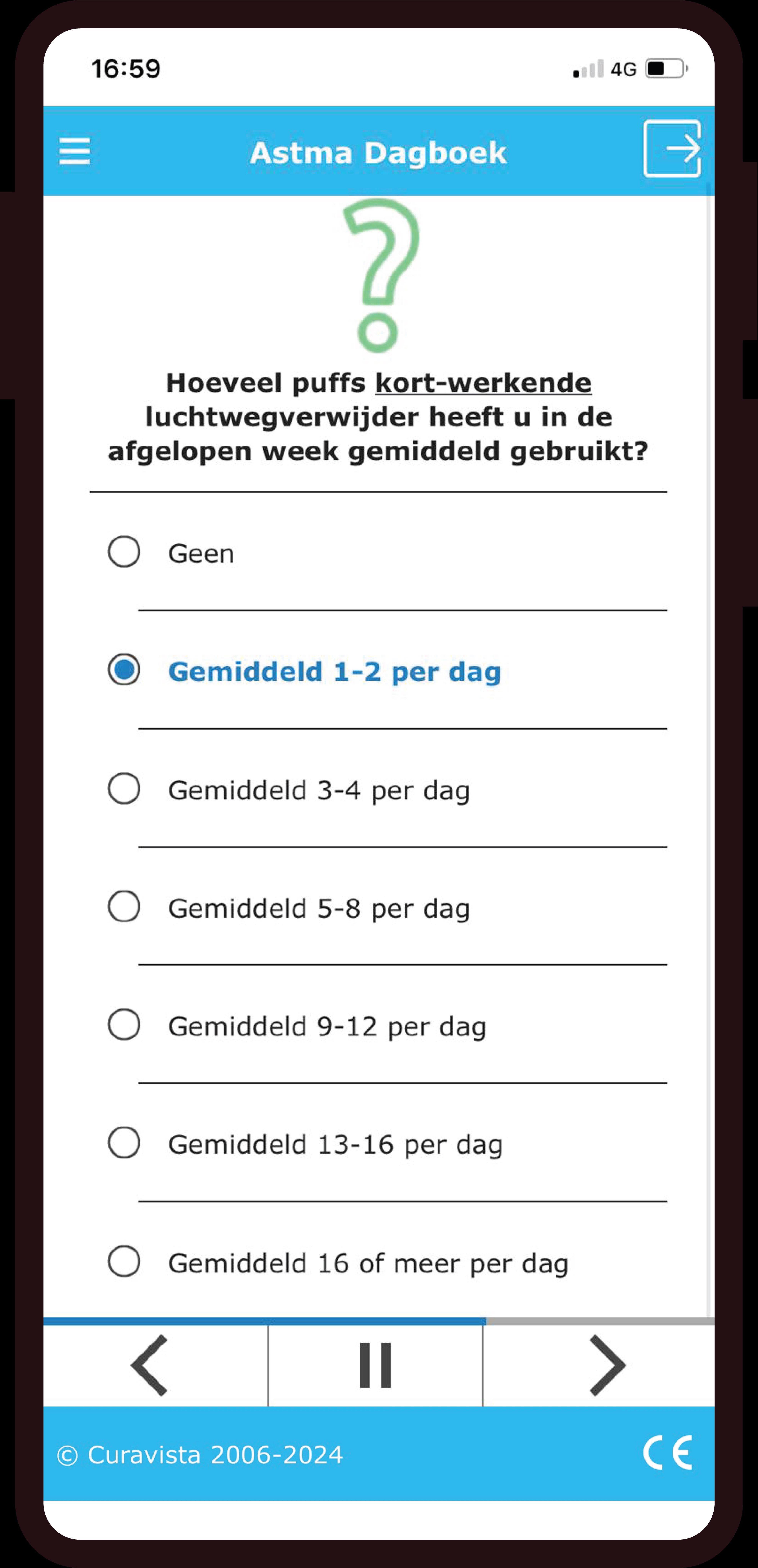

Supplement: Supplementary file 3 — Additional file 3 [file 12890_2025_3646_MOESM3_ESM.zip › Suppl. 3 Figure 1g.jpg]

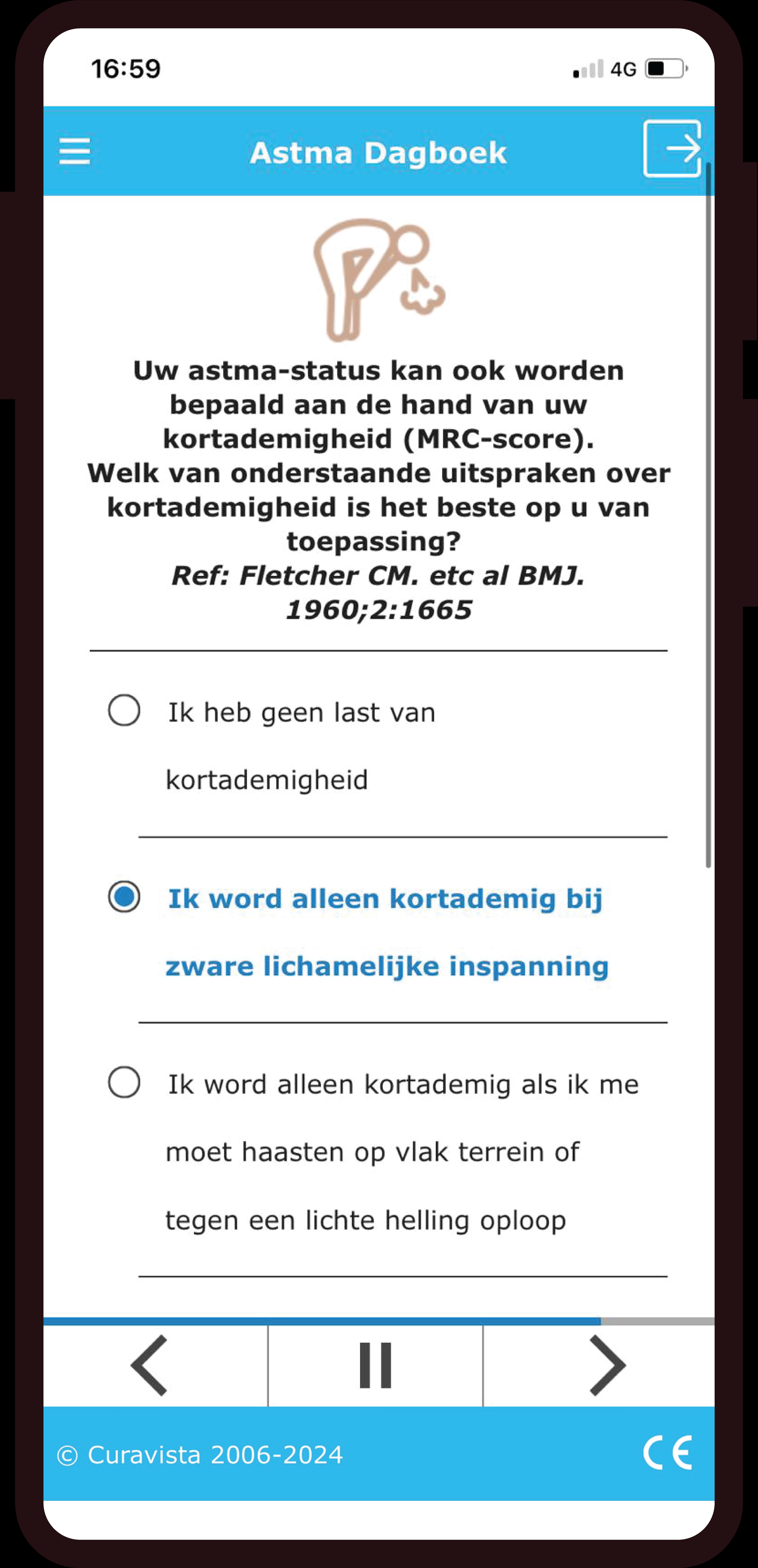

Supplement: Supplementary file 3 — Additional file 3 [file 12890_2025_3646_MOESM3_ESM.zip › Suppl. 3 Figure 1h.jpg]

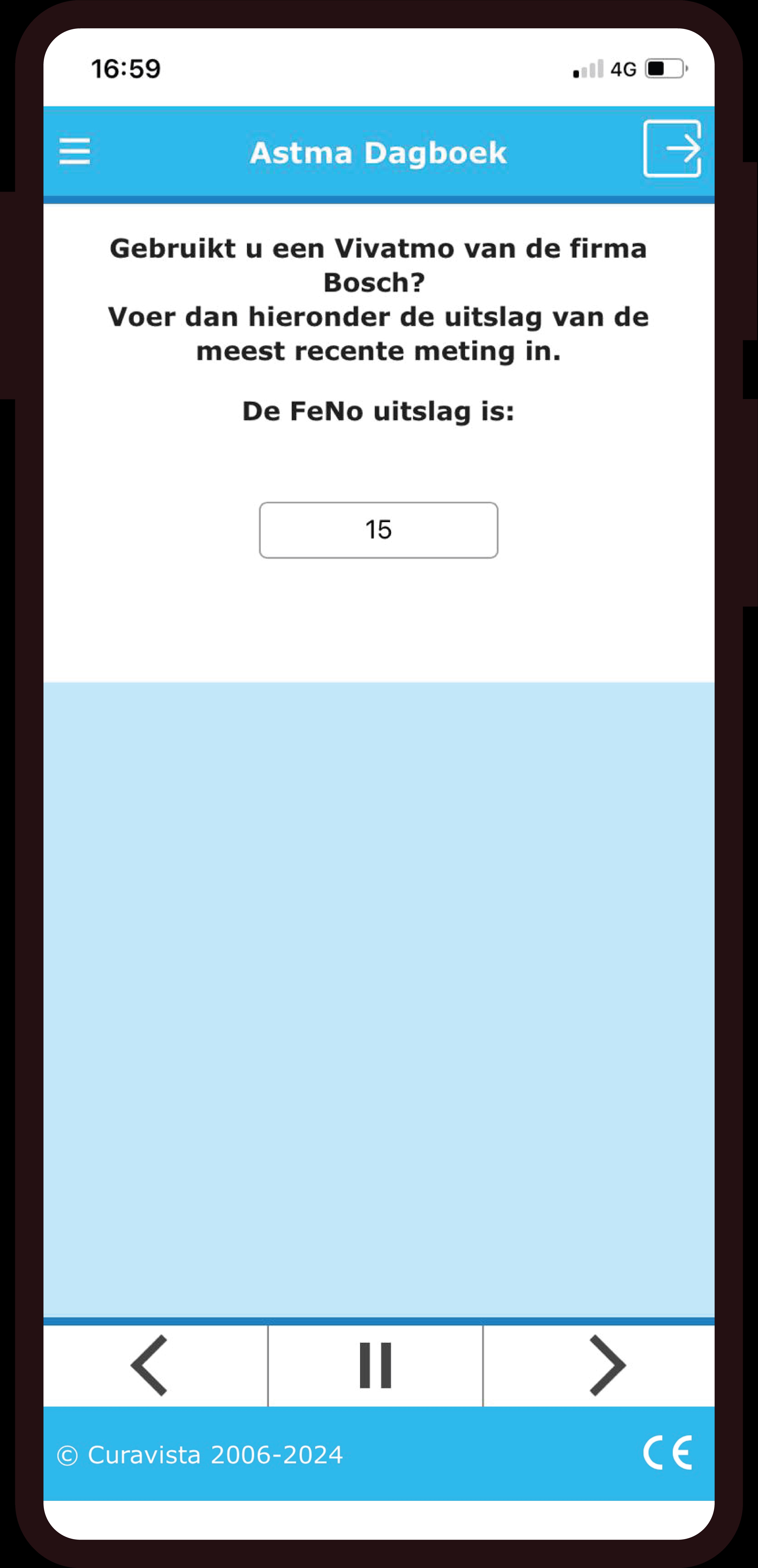

Supplement: Supplementary file 3 — Additional file 3 [file 12890_2025_3646_MOESM3_ESM.zip › Suppl. 3 Figure 1i.jpg]

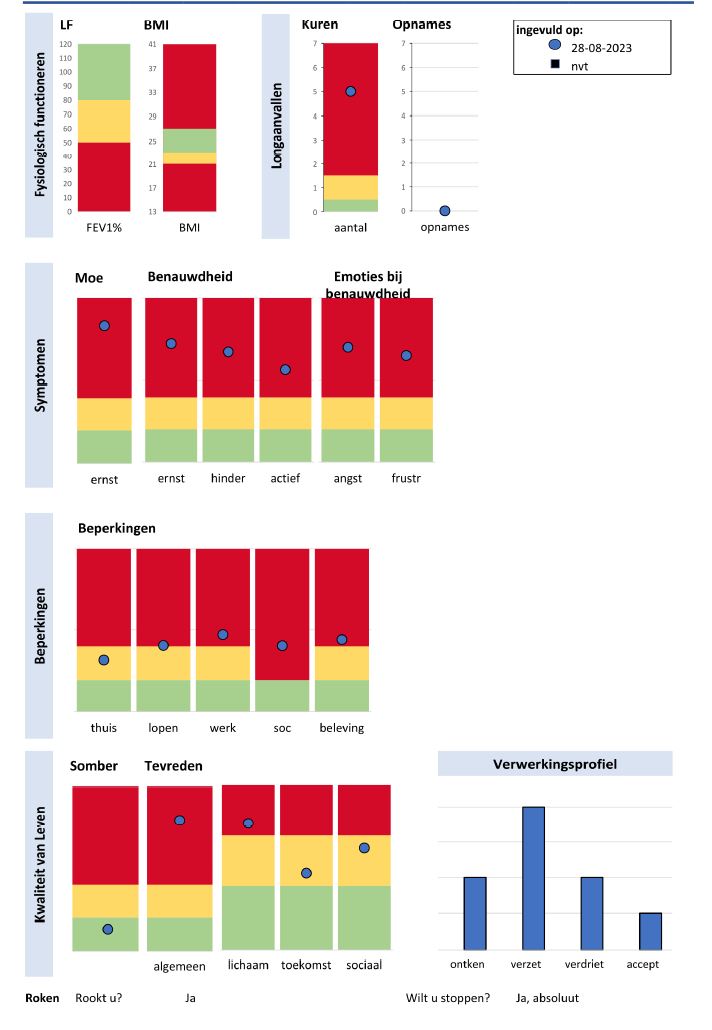

Supplement: Supplementary file 4 — Additional file 4 [file 12890_2025_3646_MOESM4_ESM.jpg]
