# Supplementary material for: The effect of a systematic multi-dimensional assessment in severe uncontrolled asthma: a literature review and protocol for an investigator-initiated, open-label, randomized-controlled trial (EXACT@home study)
Source: BMC Pulm Med. 2025 May 17;25:240. doi: 10.1186/s12890-025-03646-5 (PMC12085824; doi:10.1186/s12890-025-03646-5)
Supplement: Supplementary file 2 — Additional file 2 [file 12890_2025_3646_MOESM2_ESM.docx]

| **Alphabet treatable traits** | **Items** | **Study measurement(s)** |
| --- | --- | --- |
| **A: Asthma**  Is it asthma, what type of asthma and is it only asthma? | Asthma diagnosis according to guidelines? |  |
|  | Difficult-to-treat or severe asthma? |  |
|  | Asthma phenotype? |  |
|  | Only asthma? (e.g. COPD, EAA, ABPA, EGPA) |  |
| **B: Bronchial triggers**  Allergens and irritants causing symptoms | Active smoking? | Urine analysis: cotinine |
|  | Indication for allergens? |  |
|  | Indication for other irritants?  - passive smoking  - occupational  - hobby-related  - non-specific | Spirobank oxi: assessing change in FEV1 due to environment, hobby, work |
| **C: Comorbidities**  Comorbidities  contributing to symptoms | Only asthma? (e.g. COPD, EAA, ABPA, EGPA) |  |
|  | ENT (ear, nose, throat) problems (e.g. rhinosinusitis, nasal polyposis)? |  |
|  | GERD (gastro-esophageal reflux disease)? |  |
|  | Recurrent respiratory infections/bronchiectasis? |  |
|  | OSAS (obstructive sleep apnea syndrome)? | ESS |
|  | Dysfunctional breathing (o.a. vocal dysfunction, hyperventilation)? | NQ: subjective assessment of hyperventilation  Capillary blood gas: objective assessment of hyperventilation  Spirobank oxi: objective assessment of dysfunctional breathing |
|  | Cough hypersensitivity syndrome? |  |
|  | Psychological factors (e.g. anxiety, depression)? | HADS |
|  | Cardiovascular? |  |
|  | Active malignancy? |  |
| **D: Device**  Which device and how to use it? | Inhalation technique? | BF-Digihaler-DS: measurement of inhalation technique (flow) |
|  | Adherence? | - TAI: subjective measurement of adherence  - BF-Digihaler-DS: objective measurement of adherence  - Vivatmo me: objective measurement of adherence assessed indirectly by measuring the FeNO level |
|  | Obstacles for adherence (social, financial, societal)? |  |
| **E: Disease severity and prevention *(Dutch: ernst en preventie)***  Asthma severity and how to prevent and detect exacerbations | Determine current control | ACQ, (m)MRC, exacerbation rate, persistent obstruction (spirometry) |
|  | Identify patients with an increased risk of an exacerbation |  |
|  | Triggers for exacerbations? |  |
|  | Presence of a asthma action plan? |  |
|  | Individual definition of (severe) asthma exacerbation? |  |
| **F: Pharmacotherapy**  Which types of medication for which individual patient | Patients’ view on benefits and necessity of medication? |  |
|  | Presence of medication contraindications? |  |
|  | Presence of medication side-effects? |  |
| **G: General behavior**  How does behavior and lifestyle influence asthma and how to modify it | Physical inactivity and/or reduced exercise capacity (physical fitness)? | Marshall: subjective measurement of physical activity  Cardiowatch 287-2: objective measurement of physical activity |
|  | Obesity? |  |
|  | Stress inducing factors? |  |
|  | Impaired symptom perception/coping? |  |
|  | Passive attitude? | PAM |
|  | Family and social network? |  |
|  | Obtain insight in the patients' personal environment |  |
| **H. Help:**  Strengthen the knowledge and determine who can aid a patient in disease management | Provide education on asthma |  |
|  | (Para)medical assistance |  |
|  | Help of friends/family |  |
| **I. Individualized care plan:**  How to create and use a self-management plan for each individual patient | Developing a personalized care plan |  |
|  | Promote awareness and acceptance of individual limitations |  |
|  | Identify personal treatment goals, including willingness and potential for behavioral changes |  |
|  | Decide on treatment based on the outcomes from the EXACT@home program |  |
