# Supplementary material for: The effect of a systematic multi-dimensional assessment in severe uncontrolled asthma: a literature review and protocol for an investigator-initiated, open-label, randomized-controlled trial (EXACT@home study)
Source: BMC Pulm Med. 2025 May 17;25:240. doi: 10.1186/s12890-025-03646-5 (PMC12085824; doi:10.1186/s12890-025-03646-5)
Supplement: Supplementary file 5 — Additional file 5 [file 12890_2025_3646_MOESM5_ESM.pdf]

Proefpersoneninformatie voor deelname aan medisch-wetenschappelijk onderzoek

Versie 6.0, 19-3-2024

EXACT@home

Expertise Astma COPD traject met digitale ondersteuning

Inleiding

Geachte heer/mevrouw,

Met deze informatiebrief willen we u vragen of u wilt meedoen aan medisch-wetenschappelijk onderzoek. Meedoen is vrijwillig. Om mee te doen is wel uw schriftelijke toestemming nodig. U krijgt deze brief omdat uw astma heeft en bent benaderd door uw longarts over dit onderzoek. U leest hier om wat voor onderzoek het gaat, wat het voor u betekent, en wat de voordelen en nadelen zijn. Het is veel informatie. Wilt u de informatie rustig doorlezen en beslissen of u wilt meedoen? Als u wilt meedoen, kunt u het formulier invullen dat u vindt in bijlage D en nadien krijgt u een kopie van het ondertekende formulier mee naar huis.

Stel uw vragen

U kunt uw beslissing nemen met de informatie die u in deze informatiebrief vindt. Daarnaast raden we u aan om het volgende te doen:

- Stel vragen aan de onderzoeker die u deze informatie geeft.
- Praat met uw partner, familie of vrienden over dit onderzoek.
- Stel vragen aan de onafhankelijk deskundige, dr. B.M. van Dalen
- Lees de informatie op www.rijksoverheid.nl/mensenonderzoek

1. Algemene informatie

Het Franciscus Gasthuis & Vlietland heeft dit onderzoek opgezet. Hieronder noemen we het Franciscus Gasthuis & Vlietland steeds de ‘opdrachtgever’. De onderzoeker (een arts), voert het onderzoek uit in het Franciscus Gasthuis & Vlietland. Voor dit onderzoek zijn 138 proefpersonen nodig. De medisch-ethische toetsingscommissie MEC-U (Medical Research Ethics Committees United) heeft dit onderzoek goedgekeurd. Algemene informatie over de toetsing van onderzoek vindt u op de site: www.rijksoverheid.nl/mensenonderzoek

2. Wat is het doel van het onderzoek?

Het doel van dit onderzoek is onderzoeken en of een alternatief, intensiever zorgpad (een traject dat u doorloopt als u in het ziekenhuis komt voordat er een behandeling wordt gestart) leidt tot een betere en persoonlijkere behandeling in moeilijk behandelbaar tot ernstig astma dan het gebruikelijke, minder intensieve

zorgpad. Tevens wordt er onderzocht of dit alternatieve zorgpad leidt tot minder behandelingen met specifieke astma medicatie oftewel biologicals. In het alternatieve zorgpad maken we gebruik van onderzoeken in het ziekenhuis, een uitgebreid gesprek, vragenlijsten en digitale metingen in de thuissituatie om uiteindelijk een behandeling te kunnen kiezen. Deze behandeling kan bestaan uit het behandelen van bepaalde factoren die van invloed zijn op de symptomen van astma. Deze factoren kunnen bijvoorbeeld zijn: het juiste gebruik van de inhalatie medicatie, de correcte manier van ademhalen en/of stimuleren van beweeggedrag. Een andere behandelmogelijkheid is de behandeling met een specifiek medicijn tegen astma, een biological. Voor dit onderzoek worden patiënten gelijk verdeeld over 2 groepen: een groep die het intensievere zorgpad ondergaat en een groep die het gebruikelijke zorgpad ondergaat.

3. Wat is de achtergrond van het onderzoek?

Astma is een ziekte die veel voorkomt en klachten geeft zoals kortademigheid, hoesten en piepen. Slechts een klein deel van de patiënten met astma heeft ernstig astma. Men spreekt van ernstig astma als de astma niet onder controle is ondanks een behandeling met inhalatiemedicatie en als alle factoren die van invloed zijn aangepakt zijn.

Met dit onderzoek willen we kijken of we de behandeling van patiënten met moeilijk behandelbaar tot ernstig astma kunnen verbeteren door middel van een uitgebreide beoordeling van de factoren die van invloed zijn met behulp van een gesprek, vragenlijsten en digitale metingen in de thuissituatie om vervolgens een behandeling te kiezen die specifiek op de patiënt is gericht. Bij patiënten die deelnemen aan deze studie is de diagnose ernstig astma gesteld in een overleg tussen longartsen nadat de patiënt uitgebreid is onderzocht. De helft van de patiënten die meedoen aan deze studie ontvangt de huidige behandelmethode: een biological zoals besloten in het overleg tussen de longartsen. De andere helft ondergaat een alternatieve behandelmethode: een uitgebreide beoordeling van de factoren die van invloed zijn, onder andere met behulp van digitale metingen in de thuissituatie. Vervolgens wordt er op basis van deze informatie een geschikte behandeling gekozen. De behandeling kan bestaan uit een behandeling van factoren die van invloed zijn of een biological, of een combinatie.

4. Hoe verloopt het onderzoek?

Hoe lang duurt het onderzoek?
Doet u mee met het onderzoek? Dan duurt dat voor u in totaal ongeveer 11-12 maanden.

Stap 1: bent u geschikt om mee te doen?
Eerst bepalen we of u kunt meedoen.

U kunt met dit onderzoek mee doen als U:
- Ernstig astma heeft (diagnose gesteld in een overleg tussen longartsen)
- 18 jaar bent of ouder

U kan niet meedoen aan dit onderzoek als U:

- Op dit moment een astma aanval en/of longontsteking heeft
- Zwanger bent
- Borstvoeding geeft
- Behandeld wordt voor kanker
- Geen smartphone heeft
- Geen lichamelijke activiteit kan uitoefenen
- Recentelijk een andere astma biological heeft gehad (we nemen uw medicatiegebruik met u door)

Het is mogelijk dat er tijdens het onderzoek toevallig iets wordt ontdekt dat niet direct van belang is voor het onderzoek maar wel voor uw gezondheid of die van uw familieleden. In dit geval zal uw eigen huisarts of specialist met u bespreken wat er verder moet gebeuren. De kosten hiervan vallen onder uw eigen zorgverzekering.

Stap 2: de behandeling

Als u voor uw astma niet behandeld wordt in het Franciscus Gasthuis & Vlietland, wordt u verwezen. U zal dan voor de totale duur van het onderzoek (11-12 maanden) worden behandeld door een longarts gespecialiseerd in astma uit het Franciscus Gasthuis & Vlietland.

Voor dit onderzoek maken we 2 groepen. De eerste is de 'zorgpad' groep en de tweede de 'biological' groep. Loting bepaalt in welke groep u terecht komt. Zowel u en de onderzoeker weten in welke groep u zit.

<u>Groep 1 (alternatief, intensiever zorgpad):</u>

Nadat u bent besproken in een overleg tussen longartsen, ondergaat u gedurende 6 weken een uitgebreide beoordeling waarbij u voorafgaand een aantal vragenlijsten invult en 4 of 5 digitale apparaten mee naar huis krijgt, bestaande uit een longfunctiemeter, 2 beweegmeters (een armband en een borstband), een digitale puffer en eventueel een ontstekingsmeter. Daarnaast krijgt u toegang tot de Gezondheidsmeter (een digitale omgeving van Curavista).

- De longfunctiemeter meet de mate van luchtwegvernauwing en gebruikt u elke dag voor inname van uw inhalatiemedicatie en tijdens momenten van kortademigheid.
- De eerste beweegmeter (armband) is een armband die beweging, slaap, ademhaling, hartslag, zuurstofgehalte en temperatuur meet en een hartfilmpje maakt. Deze armband draagt u de hele dag. U kunt zelf uw resultaten inzien in de app van deze beweegmeter. Na 5 weken krijgt u gedurende 1 week een tweede beweegmeter (borstband) mee die u de hele dag om de borst draagt. Deze borstband meet alleen de beweging.
- De digitale puffer zal (een deel van) uw eigen puffer(s) vervangen. De digitale puffer bevat standaard, erkende astma medicatie, namelijk budesonide/formoterol, wat overeenkomt met of gelijk is aan (een deel van) uw eigen astma medicatie. Eigen aanvullende langwerkende medicatie, kortwerkende medicatie en/of vernevelingen kunnen indien nodig worden gecontinueerd. De digitale puffer meet gedurende de hele studie (11-12 maanden) uw gebruik van de inhalatiemedicatie, waaronder het moment en de techniek van inname. De digitale puffer is vergelijkbaar qua vorm met een normale puffer. U kunt zelf uw resultaten inzien in de app van de digitale puffer.

- U krijgt alleen een ontstekingsmeter mee, indien u een verhoogde ontstekingswaarde (FeNO) in uw luchtwegen heeft. Deze gebruikt u elke dag samen met de longfunctiemeter om de mate van luchtwegontsteking te meten.
- In de Gezondheidsmeter kunt u de resultaten van de apparaten (behalve beide beweegmeters en digitale puffer) inzien en kan u in een astma dagboek invullen hoe het met u gaat.

Na 6 weken krijgt u een uitgebreid gesprek met de longconsulent en longarts waarin een aantal vragen worden gesteld en ook de uitslagen van de vragenlijsten en digitale thuismetingen worden meegenomen. Hierna wordt een behandeling gekozen. Deze behandeling kan bestaan uit een biological en/of behandeling van de factoren die van invloed zijn. Vervolgens wordt u t/m 11-12 maanden geobserveerd om te onderzoeken of de gekozen behandeling effectief is. Indien nodig zal gedurende deze 11-12 maanden uw behandeling worden aangepast.

De tweede beweegmeter (borstband) levert u na 1 week gebruiken weer in. De longfunctiemeter, ontstekingsmeter en eerste beweegmeter (armband) gebruikt u in totaal 12 weken en levert u daarna in. De digitale puffer en de Gezondheidsmeter gebruikt u tot het einde van de studie (11-12 maanden).

<u>Groep 2 (gebruikelijke, minder intensieve zorgpad)</u>
U ontvangt de standaard zorg voor ernstig astma. U krijgt gelijk een biological zoals besloten in het overleg tussen longartsen en u wordt in het gebruik daarvan begeleid. U doet 11-12 maanden mee met het onderzoek om te kijken of de biological effectief is. Tevens krijgt u voor 11-12 maanden een digitale puffer die een deel van uw eigen puffer(s) vervangt, waardoor wij uw inhalatiegebruik kunnen bijhouden. U kunt zelf uw gebruik van inhalatiemedicatie niet inzien. Ook krijgt u voor 11-12 maanden toegang tot de Gezondheidsmeter (een digitale omgeving van Curavista) zodat u in een astma dagboek kan invullen hoe het met u gaat. U wordt gedurende 11-12 maanden geobserveerd om te onderzoeken of de gekozen biological effectief is. Indien nodig zal gedurende deze 11-12 maanden uw behandeling worden aangepast.

Stap 3: onderzoeken en metingen
Voor het onderzoek is het nodig dat u 5-6 keer in het ziekenhuis komt in 11-12 maanden. Daarnaast zijn er nog 2 bel of video-afspraken. Een bezoek duurt ongeveer een half uur tot een uur. Dat lijkt veel maar is bij de analyse en behandeling van moeilijk behandelbaar tot ernstig astma gebruikelijk en overeenkomstig met de gebruikelijke zorg.
We doen de volgende onderzoeken:

- Lichamelijk onderzoek: Dit is standaard zorg en geen specifiek onderdeel van de studie. De onderzoeker luistert naar uw longen en uw hartslag en uw zuurstofgehalte, ademhaling en bloeddruk worden gemeten.
- Onderzoek van uw bloed en urine: Met het bloed en urineonderzoek meten we o.a. blootstelling aan stoffen, ontstekingswaardes, bloedgehalte, cellen van het immuunsysteem, antistoffen en het zuurstof- en koolzuurgehalte. De bloedafname in standaard zorg en geen specifiek onderdeel van de studie. Alles bij elkaar nemen we ongeveer 4 tot 6 buisjes bloed bij u af (4-6 ml bloed per buisje). Daarnaast krijgt aan het begin van de studie eenmalig een vingerprik om een paar druppels bloed (100 μl) af te nemen. De arts kan indien nodig beslissen meer bloedonderzoek in te zetten.

- U vult een aantal vragenlijsten in verspreid over de bezoeken (14 verschillende in groep 1 en 13 verschillende in groep 2, waarvan 2 niet verplicht zijn voor de studie): De vragen gaan o.a. over klachten die u ervaart van uw ziekte, hoe u met uw ziekte en klachten omgaat, uw kwaliteit van leven en welzijn, hoe tevreden u bent met de geleverde zorg, hoe u uw inhalatiemedicatie inneemt, wat uw lichamelijke activiteit is en over kennis, vaardigheden en vertrouwen in het kunnen managen van uw eigen gezondheid of ziekte. *Duur totaal aantal vragenlijsten: 45-50 minuten in groep 1 en 30 minuten in groep 2*
- Longfunctie en luchtwegontsteking: Dit is standaard zorg en geen specifiek onderdeel van de studie. Er wordt een longfunctie gemeten op de longfunctie afdeling. Hiermee wordt gekeken of er vernauwing van de luchtwegen aanwezig is. Ook wordt de mate van ontsteking in de luchtwegen gemeten op de longfunctie afdeling. *Duur: 15-45 minuten (afhankelijk van hoe uitgebreid het onderzoek is).*
- Röntgenfoto van de longen: Dit is standaard zorg en geen specifiek onderdeel van de studie. Er wordt aan het begin van de studie een longfoto gemaakt indien u > 6 maanden geleden voor het laatst een longfoto heeft laten maken.
- Elektronische neus: Middels dit apparaat wordt de uitgeademde lucht onderzocht op deeltjes die mogelijk van belang zijn voor uw astma. *Duur: 2-3 minuten.*

Tijdens de visites kan door de longarts besloten worden om meer aanvullend onderzoek in te zetten dan hierboven beschreven is omdat dit voor u behandeling noodzakelijk is.

Tijdens de digitale thuismetingen wordt er door de zorgverlener of onderzoeker niet gecontroleerd op afwijkende waardes. Wel worden de waardes achteraf beoordeeld tijdens de afspraken bij de zorgverlener.

In bijlage C staat uitgebreid beschreven welke handelingen we doen bij ieder bezoek.

Wat is er anders dan bij gewone zorg?
In beide groepen is er 1 extra afspraak naast de gewone zorg bestaande uit een afspraak in het ziekenhuis. De controles die bij dit onderzoek horen, komen in de plaats van deze controles bij uw eigen arts.

5. Welke afspraken maken we met U?

We willen graag dat het onderzoek goed verloopt. Daarom maken we de volgende afspraken met u

- U gebruikt de apparaten op de manier die de onderzoeker u heeft uitgelegd.
- U gebruikt de digitale puffer in plaats van uw eigen puffer(s).
- U doet tijdens dit onderzoek niet ook nog mee aan een ander medisch-wetenschappelijk onderzoek.
- U komt naar iedere afspraak.
- U noteert in de Gezondheidsmeter als u een longaanval heeft en/of opgenomen bent in een ziekenhuis.
- U draagt de deelnemerskaart van het onderzoek bij u. Bijvoorbeeld in uw portemonnee. Hierop staat dat u meedoet aan dit onderzoek en wie men op de hoogte moet stellen bij een longaanval en/of ziekenhuisopname en met wie men contact kan opnemen. Laat deze kaart zien als u bij een andere arts dan uw behandeld longarts in het Franciscus Gasthuis & Vlietland komt.

- U neemt contact op met de onderzoeker in deze situaties:
    - U wilt andere inhalatiemedicatie, prednison of antibiotica gaan gebruiken.
    - U wordt in een ziekenhuis opgenomen of behandeld.
    - U krijgt plotseling problemen met uw gezondheid.
    - U wilt niet meer meedoen met het onderzoek.
    - Uw telefoonnummer, adres of e-mailadres verandert.

Mag u of uw partner zwanger worden of borstvoeding geven tijdens het onderzoek?
Vrouwen die zwanger zijn en vrouwen die borstvoeding geven, kunnen niet meedoen aan dit onderzoek. Wordt u toch zwanger tijdens het onderzoek of gaat u borstvoeding geven? Laat dit dan meteen weten aan de onderzoeker. In dat geval zal u niet meer mee kunnen doen aan de studie, maar wordt de behandeling wel voorgezet.

Toch zwanger of borstvoeding geven tijdens het onderzoek?
Wordt u toch zwanger tijdens het onderzoek of gaat u borstvoeding geven? Laat dit dan meteen weten aan de onderzoeker. In dat geval zal u niet meer mee kunnen doen aan de studie, maar wordt de behandeling wel voorgezet.

6. Van welke bijwerkingen, nadelige effecten of ongemakken kunt u last krijgen?

Er worden geen risico's verwacht van de beweegmeters. Het blazen van een longfunctie of het meten van luchtwegontsteking en het meten van deeltjes in uitgeademde lucht met de elektronische neus kan mogelijk leiden tot een licht gevoel in het hoofd of flauwvallen door hyperventilatie. Bloedafnames kunnen pijn doen of een bloeduitstorting geven. Voor het gebruik van de digitale puffer wordt uw eigen inhalatiemedicatie omgezet naar eerste keus inhalatiemedicatie (budesonide/formoterol). Het is mogelijk dat de digitale puffer en/of de inhalatiemedicatie in de digitale puffer (budesonide/formoterol) uiteindelijk niet geschikt voor u blijkt. In dit geval kunt u de digitale puffer niet meer gebruiken en zal de medicatie worden aangepast.

Mogelijke bijwerkingen van de standaard, erkende astma medicatie (budesonide/formoterol) in de digitale puffer zijn:
Vaak: kunnen optreden bij 1 op 10 mensen

- Hartkloppingen, trillen of schudden. Als deze effecten optreden, zijn ze meestal licht en verdwijnen ze meestal als u doorgaat met het gebruik van De BF-Digihaler;
- Spruw (een schimmelinfectie) in de mond. Dit komt waarschijnlijk minder vaak voor als u na het gebruik van uw geneesmiddel uw mond met water spoelt;
- Lichte keelpijn, hoesten en een hese stem;
- Hoofdpijn;
- Pneumonie (longinfectie)

Soms: kunnen optreden bij 1 op 100 mensen

- Rusteloos gevoel, nerveus, angstig of boos;
- Verstoorde slaap;
- Duizeligheid;
- Misselijkheid (ziek voelen);
- Een snelle hartslag;
- Blauwe plekken op de huid;
- Spierkrampen;
- Wazig zien.

Bijwerkingen die zelden of zeer zelden optreden staan beschreven in de bijsluiter van de digitale puffer. Doet u mee aan het onderzoek, dan krijgt u een bijsluiter mee bij de digitale puffer. Tevens krijgt u uitleg mee over de longfunctiemeter, ontstekingsmeter, beweegmeters en digitale omgeving.

7. Wat zijn de voordelen en nadelen als u meedoet aan het onderzoek?

Meedoen aan het onderzoek kan voordelen en nadelen hebben. Hieronder zetten we ze op een rij. Denk hier goed over na, en praat erover met anderen.

Pas aan het begin van het onderzoek wordt duidelijk of de patiënt in groep 1 of 2 wordt ingedeeld. Het voordeel van groep 1 (zorgpad) is een uitgebreide beoordeling en mogelijk een persoonsgerichte en effectieve behandeling. Het nadeel van groep 1 is dat de mogelijke behandeling met biologicals kortdurend wordt uitgesteld. Het is ook mogelijk dat de biological na de beoordeling niet meer noodzakelijk blijkt te zijn. Het voordeel van groep 2 (rechtstreeks biological) is dat er direct met een mogelijk effectieve biological wordt gestart. Het nadeel van groep 2 is dat de biological eventueel te snel gestart wordt, terwijl verder onderzoek noodzakelijk is.

Verdere nadelen van meedoen aan dit onderzoek kunnen zijn:

- U kunt last krijgen van mogelijke ongemakken zoals beschreven in paragraaf 6.
- Meedoen aan het onderzoek kost u extra tijd. U krijgt 1 extra afspraak in het ziekenhuis vergeleken met de standaard behandeling bij moeilijk behandelbaar tot ernstig astma.
- Voor het onderzoek moet u naar het Astma Expertise centrum van het Franciscus Gasthuis & Vlietland komen in plaats van uw eigen ziekenhuis.
- U moet zich houden aan de afspraken die horen bij het onderzoek.

8. Wanneer stopt het onderzoek?

De onderzoeker laat het u weten als er nieuwe informatie over het onderzoek is die belangrijk voor u is. De onderzoeker vraagt u daarna of u blijft meedoen.

In deze situaties stopt voor u het onderzoek:

- Alle onderzoeken volgens het schema in paragraaf 4 zijn voorbij.
- Het einde van het hele onderzoek is bereikt (u heeft 11-12 maanden meegedaan).
- U bent zwanger geworden.
- U wilt zelf stoppen met het onderzoek. Dat mag op ieder moment. Meld dit dan meteen bij de onderzoeker. U hoeft er niet bij te vertellen waarom u stopt. U krijgt dan weer de gewone behandeling voor uw astma.
- De onderzoeker vindt het beter voor u om te stoppen.
- Een van de volgende instanties besluit dat het onderzoek moet stoppen:
  - Franciscus Gasthuis & Vlietland
  - de overheid, of
  - de medisch-ethische commissie die het onderzoek beoordeelt.

Het hele onderzoek is afgelopen als alle deelnemers 11-12 maanden hebben mee gedaan.

Als u niet wilt meedoen of wilt stoppen met het onderzoek
U beslist zelf of u meedoet aan het onderzoek. Deelname is vrijwillig. Wilt u niet meedoen? Dan krijgt u de gewone behandeling voor uw astma.

Als u wel meedoet, kunt u zich altijd bedenken en toch stoppen, ook tijdens het onderzoek. U wordt dan weer op de gebruikelijke manier behandeld voor uw astma. U hoeft niet te zeggen waarom u stopt. Wel moet u dit direct melden aan de onderzoeker. De gegevens die tot dat moment zijn verzameld, worden gebruikt voor het onderzoek.

9. Wat gebeurt er na het onderzoek?

Na het onderzoek wordt de ingezette behandeling (indien effectief) voorgezet en krijgt u de gebruikelijke zorg voor moeilijk behandelbaar tot ernstig astma. U wordt (indien u dit ook wenst) terug verwezen naar uw vorige longarts, als u voorafgaand aan het onderzoek in een ander ziekenhuis dan het Franciscus Gasthuis & Vlietland voor uw astma werd behandeld.

Kunt u de apparaatjes en Gezondheidsmeter blijven gebruiken?
De digitale puffer kunt u na het onderzoek niet blijven gebruiken. Na het onderzoek wordt de digitale puffer omgezet naar een normale puffer met dezelfde werking en medicatie. U kunt wel gebruik blijven maken van de Gezondheidsmeter.

Krijgt u de resultaten van het onderzoek?
Ongeveer 2 jaar nadat het onderzoek is afgerond laat de onderzoeker u weten wat de belangrijkste uitkomsten zijn van het onderzoek. Wilt u dit niet weten? Zeg dat dan tegen de onderzoeker. Hij/zij zal het u dan niet vertellen.

10. Wat doen we met uw gegevens?

Doet u mee met het onderzoek? Dan geeft u ook toestemming om uw gegevens te verzamelen, gebruiken en bewaren.

Welke gegevens bewaren we?

We bewaren deze gegevens:

- uw naam
- uw geslacht
- uw adres
- uw geboortedatum
- gegevens over uw gezondheid
- (medische) gegevens en resultaten die we tijdens het onderzoek verzamelen

Waarom verzamelen, gebruiken en bewaren we uw gegevens en resultaten?

We verzamelen, gebruiken en bewaren uw gegevens en resultaten om de vragen van dit onderzoek te kunnen beantwoorden. En om de resultaten te kunnen publiceren. Gegevens en resultaten kunnen worden gebruikt door de onderzoeker(s) en bedrijven die de onderzoeker(s) helpen bij het uitvoeren van de studie en/of het analyseren van onderzoeksgegevens.

Voor het belang van dit onderzoek en uw behandeling worden uw gegevens opgevraagd bij uw vorige longarts, huisarts en eigen apotheek. Tevens wordt uw informatie over o.a. ziekenhuisopnames, medicatie voorschriften, poli- en huisartsbezoeken gerelateerd aan astma die plaatsvinden tijdens de totale duur van het onderzoek (11-12 maanden) opgevraagd.

Voor het gebruik van de Gezondheidsmeter, de app van eerste beweegmeter (armband), de app van de digitale puffer en de elektronische neus worden uw gecodeerde, gepseudonimiseerde gegevens opgeslagen in een beveiligde Europese Cloud, zodat de onderzoeker en zorgverlener de gegevens kunnen inzien en gebruiken. De producenten van deze applicaties/apparaten hebben alleen toegang tot gecodeerde, gepseudonimiseerde gegevens.

Voor de ontwikkeling, verbetering en het naar de markt brengen van medische hulpmiddelen en producten worden gecodeerde, gepseudonimiseerde gegevens gedeeld met de producenten van de Digitale inhalator (Teva), de elektronische neus (Breathomix) en een vragenlijst die kennis, vaardigheden en vertrouwen in het kunnen managen van de eigen gezondheid of ziekte meet (PAM, Insignia Health).

In het toestemmingformulier geeft u aan of bovenstaande goed vindt. Als u dit niet goed vindt, kunt u niet meedoen aan dit onderzoek.

Hoe beschermen we uw privacy?

Om uw privacy te beschermen geven wij uw gegevens en resultaten een code. Op al uw gegevens en resultaten zetten we alleen deze code. De sleutel van de code bewaren we op een beveiligde plek in de onderzoekslocatie (Franciscus Gasthuis & Vlietland). Als we uw gegevens en resultaten verwerken, gebruiken we steeds alleen die code. Ook in rapporten en publicaties over het onderzoek kan niemand terughalen dat het over u ging.

Wie kunnen uw gegevens zien?
Sommige personen kunnen wel uw naam en andere persoonlijke gegevens zonder code inzien. Dit kunnen gegevens zijn die speciaal voor dit onderzoek zijn verzameld, maar ook gegevens uit uw medisch dossier. Dit zijn mensen die controleren of de onderzoekers het onderzoek goed en betrouwbaar uitvoeren. Deze personen kunnen bij uw gegevens komen:

- Leden van de commissie die de veiligheid van het onderzoek in de gaten houden.
- Een controleur die voor de onderzoeker(s) van het onderzoek werkt.
- Nationale en internationale toezichthoudende autoriteiten. Bijvoorbeeld de Inspectie Gezondheidszorg en Jeugd of de EMA (European Medicines Agency).

Deze personen houden uw gegevens geheim. Wij vragen u voor deze inzage toestemming te geven. De Inspectie Gezondheidszorg en Jeugd kan zonder uw toestemming uw gegevens inzien.

Hoe lang bewaren we uw gegevens en resultaten?
We bewaren uw verzamelde gegevens 15 jaar op de onderzoekslocatie (Franciscus Gasthuis & Vlietland).

Mogen we uw gegevens en lichaamsmateriaal gebruiken voor ander onderzoek?
Uw verzamelde gegevens kunnen ook van belang zijn voor ander wetenschappelijk onderzoek op het gebied van astma. Daarvoor zullen uw 15 jaar worden bewaard op de onderzoekslocatie (Franciscus Gasthuis & Vlietland). In het toestemmingformulier geeft u aan of u dit goed vindt. Geeft u geen toestemming? Dan kunt u nog steeds meedoen met dit onderzoek. U krijgt dezelfde zorg.

Wat gebeurt er met onverwachte ontdekkingen?
Tijdens het onderzoek kunnen we toevallig iets vinden dat niet direct van belang is voor het onderzoek maar wel voor uw gezondheid. De onderzoeker neemt dan contact op met uw huisarts of specialist. U bespreekt dan met uw huisarts of specialist wat er moet gebeuren. De kosten hiervan vallen onder uw eigen zorgverzekering. U geeft met het formulier toestemming voor het informeren van uw huisarts of specialist.

Kunt u uw toestemming voor het gebruik van uw gegevens weer intrekken?
U kunt uw toestemming voor het gebruik van uw gegevens op ieder moment intrekken. Dit geldt voor het gebruik in dit onderzoek en voor het gebruik in ander onderzoek. Maar let op: trekt u uw toestemming in, en hebben onderzoekers dan al gegevens verzameld voor een onderzoek? Dan mogen zij deze gegevens nog wel gebruiken.
Wilt u meer weten over uw privacy?

- Wilt u meer weten over uw rechten bij de verwerking van persoonsgegevens? Kijk dan op www.autoriteitpersoonsgegevens.nl.
- Heeft u vragen over uw rechten? Of heeft u een klacht over de verwerking van uw persoonsgegevens? Neem dan contact op met degene die verantwoordelijk is voor de verwerking van uw persoonsgegevens. Voor uw onderzoek is dat:
    - Franciscus Gasthuis & Vlietland. Zie bijlage A voor contactgegevens, en website.

- Als u klachten heeft over de verwerking van uw persoonsgegevens, raden we u aan om deze eerst te bespreken met het onderzoeksteam. U kunt ook naar de Functionaris Gegevensbescherming van de onderzoekslocatie (Franciscus Gasthuis & Vlietland) gaan. Of u dient een klacht in bij de Autoriteit Persoonsgegevens.

Waar vindt u meer informatie over het onderzoek?
Op de volgende website(s) vindt u meer informatie over het onderzoek: *toetsingonline.nl*. Daarin zijn geen gegevens opgenomen die naar u herleidbaar zijn. Na het onderzoek kan de website een samenvatting van de resultaten van dit onderzoek tonen. U vindt het onderzoek door te zoeken op ‘NL79996.100.22’.

11. Krijgt u een vergoeding als u meedoet aan het onderzoek?

De studie betaalt de digitale apparaatjes, applicaties en medicatie in de digitale puffer. De onderzoeken, astma behandeling en bezoeken aan het ziekenhuis vallen onder de standaard zorg en worden door uw verzekering betaald (houd rekening met uw eigen risico). U wordt niet betaald voor het meedoen aan dit onderzoek. Wel krijgt u een vergoeding voor de parkeerkosten in vorm van een uitrijkaart bij elk bezoek aan het ziekenhuis. Daarnaast ontvangt u aan het einde van uw deelname aan het onderzoek een cadeaukaart ter waarde van 50 euro als tegemoetkoming voor reiskosten.

12. Bent u verzekerd tijdens het onderzoek?

Voor iedereen die meedoet aan dit onderzoek is een verzekering afgesloten. De verzekering betaalt voor schade door het onderzoek. Maar niet voor alle schade. In bijlage B vindt u meer informatie over de verzekering en de uitzonderingen. Daar staat ook aan wie u schade kunt melden.

13. We informeren uw huisarts, vorige longarts en eigen apotheek

De onderzoeker stuurt uw huisarts, uw vorige longarts en eigen apotheek een brief om te laten weten dat u meedoet aan het onderzoek. Dit is voor uw eigen veiligheid. Als u dit niet goed vindt, kunt u niet meedoen aan dit onderzoek.

14. Heeft u vragen?

Vragen over het onderzoek kunt u stellen aan de arts-onderzoeker. Wilt u advies van iemand die er geen belang bij heeft? Ga dan naar onafhankelijke arts (bijlage A). Hij weet veel over het onderzoek, maar werkt niet mee aan dit onderzoek.

Heeft u een klacht? Bespreek dit dan met de onderzoeker of de arts die u behandelt. Wilt u dit liever niet? Ga dan naar klachtenfunctionaris van het ziekenhuis. In bijlage A staat waar u die kunt vinden.

15. Hoe geeft u toestemming voor het onderzoek?

U kunt eerst rustig nadenken over dit onderzoek. Daarna vertelt u de onderzoeker of u de informatie begrijpt en of u wel of niet wilt meedoen. Wilt u meedoen? Dan vult u het toestemmingsformulier in dat u bij deze informatiebrief vindt. U en de onderzoeker krijgen allebei een getekende versie van deze toestemmingsverklaring.

Dank voor uw tijd.

16. Bijlagen bij deze informatie

A. Contactgegevens Franciscus Gasthuis & Vlietland – pagina 11
B. Informatie over de verzekering – pagina 12
C. Schema onderzoekshandelingen – pagina 13 t/m 14
D. Toestemmingsformulier(en) – pagina 15 t/m 16

Bijlage A: contactgegevens Franciscus Gasthuis & Vlietland

Als u nog vragen heeft over dit onderzoek, neem dan contact op met de arts-onderzoeker:

De (hoofd)onderzoekers:
Dr. J.C.C.M. In ’t Veen, Longarts
Telefoonnummer: tel: 010 - 461 61 49 (tijdens kantooruren)

Dr. G.J. Braunstahl, Longarts
Telefoonnummer: 010 - 461 61 49 (tijdens kantooruren)

De arts-onderzoeker longziekten:
Drs. L. (Lotte) Bult
Telefoonnummer: 06 – 12 28 00 07 (tijdens kantooruren)
E-mail: exact_home@franciscus.nl

De onafhankelijk arts:
Dr. B.M. Van Dalen, cardioloog
Telefoonnummer: 010 - 461 61 62 (tijdens kantooruren)

Buiten kantooruren kunt u met het algemene nummer van het ziekenhuis bellen:
Franciscus Gasthuis: 010-461 61 61
Franciscus Vlietland: 010-893 93 93
en vragen naar de dienstdoende arts van de longziekten.

Klachten:
Deze studie wordt uitgevoerd met toestemming van de Raad van Bestuur van dit ziekenhuis.
Het Franciscus Gasthuis & Vlietland vindt het belangrijk dat patiënten, proefpersonen en bezoekers tevreden zijn. Toch kan het gebeuren dat u niet tevreden bent en een klacht wilt indienen. In dat geval kunt u het beste eerst praten met de arts-onderzoeker of uw behandelend longarts. Als u dat liever niet doet, kunt u ook contact opnemen met de cliëntvertrouwenspersoon van het ziekenhuis. Dit kan zowel telefonisch als door het invullen van het online klachtenformulier.

Franciscus Gasthuis & Vlietland (en alle buitenpoli’s):
Telefoonnummer: 010 – 893 4125

Digitaal via www.franciscus.nl/klacht (voor alle locaties)

Functionaris Gegevensbescherming (alle locaties):
E-mail: fg@franciscus.nl

Bijlage B: informatie over de verzekering

Voor iedereen die meedoet aan dit onderzoek, heeft het Franciscus Gasthuis & Vlietland een verzekering afgesloten. De verzekering dekt schade door deelname aan het onderzoek. Dit geldt voor schade tijdens het onderzoek of binnen vier jaar na het einde ervan. Schade moet u binnen die vier jaar aan de verzekeraar hebben gemeld.

De verzekering dekt niet alle schade. Onderaan deze tekst staat in het kort welke schade niet wordt gedekt. Deze bepalingen staan in het Besluit verplichte verzekering bij medisch-wetenschappelijk onderzoek met mensen. Dit besluit staat op www.ccmo.nl/, de website van de Centrale Commissie Mensgebonden Onderzoek (zie 'Bibliotheek' en dan 'Wet- en regelgeving').

Bij schade kunt u contact opnemen met de cliëntvertrouwenspersoon van het Franciscus Gasthuis & Vlietland. Vermeld hierbij aan welke wetenschappelijke studie u deelneemt en waar deze verzekerd is.

Bereikbaarheid cliëntvertrouwenspersoon:
Franciscus Gasthuis & Vlietland (en buitenpoli's)
Tel.: 010 – 893 4125

De verzekeraar van het onderzoek is:
Naam: Onderlinge Waarborgmaatschappij Centramed B.A.
Adres: Postbus 7374, 2701 AJ Zoetermeer
Telefoon: 070 301 7070
E-mail: schade@centramed.nl

De verzekering biedt een dekking van € 650.000,- per proefpersoon en € 5.000.000,- voor het hele onderzoek en € 7.500.000,- per jaar voor alle onderzoeken van dezelfde opdrachtgever.

De verzekering dekt de volgende schade **niet**:

- schade door een risico waarover u in de schriftelijke informatie bent ingelicht. Dit geldt niet als het risico zich ernstiger voordoet dan was voorzien of als het risico heel onwaarschijnlijk was;
- schade aan uw gezondheid die ook zou zijn ontstaan als u niet aan het onderzoek had meegedaan;
- schade door het niet (volledig) opvolgen van aanwijzingen of instructies;
- schade aan uw nakomelingen, als gevolg van een negatief effect van het onderzoek op u of uw nakomelingen;
- schade door een bestaande behandelmethode bij onderzoek naar bestaande behandelmethoden.

Bijlage C: Schema onderzoekshandelingen

Visite	Tijd	Afspraak	Activiteit		
Visite 1: start studie	Week 0	Arts-onderzoeker	Toestemmingsformulier ondertekenen en verdeling in een van beide groepen		
				Groep 1 (zorgpad)	***Groep 2 (biologicals)***
			Verzamelen van medische gegevens	X	X
			Lichamelijk onderzoek	X	X
			Vragenlijsten	X	X
			Röntgenfoto van de longen	X (alleen indien > 6 maanden geleden)	X (alleen indien > 6 maanden geleden)
			Bloed prikken en urine inleveren	X	X
			Meting met elektronische neus	X	X
			Uitleg en installatie applicaties	X	X
			Uitleg en uitgifte longfunctiemeter, ontstekingsmeter en eerste beweegmeter (armband)	X	
			Start Digitale omgeving (Gezondheidsmeter)	X	X
		Dagbehandeling	Start biological		X
		Apotheek	Uitleg en uitgifte digitale puffers	X	X
Visite 2	+/- Week 5	Longconsulent	Uitgebreid gesprek over uw ziekte	X	
			Uitleg en uitgifte tweede beweegmeter (borstband)	X	
			Extra vragenlijst	X	
		Afspraak fysiotherapeut	Onderzoek beweeggedrag en uithoudingsvermogen	X	
Visite 3	+/- Week 6	Afspraak arts-onderzoeker	Vragenlijst	X	X
		Longfunctie	Uitgebreide longfunctie en meten luchtwegontsteking bij longfunctieafdeling	X	X
		Afspraak longconsulent	Inleveren tweede beweegmeter (borstband)	X	
			Lichamelijk onderzoek	X	
			Vragenlijsten	X	
			Uitgebreid gesprek over bijdragende kenmerken	X	
		Afspraak longarts	Start behandeling van bijdragende factor(en) en/of biological	X	
			Beoordelen effect biological		X
			Bloed prikken		X
		Apotheek	Inleveren en uitgifte digitale puffers	X	X
Visite 4	+/- Week 12	Afspraak longarts	Inleveren alle apparaatjes, behalve digitale puffer	X	
			Beoordelen effect behandeling	X	X
			Vragenlijsten	X	X
		Afspraak arts-onderzoeker	Meting met elektronische neus	X	X
		Apotheek	Inleveren en uitgifte digitale puffers	X	X

Visite 5	+/- Maand 6	Afspraak arts-onderzoeker	Vragenlijsten	X	X
			Meting met elektronische neus	X	X
		Afspraak longarts	Beoordelen effect behandeling	X	X
		Longfunctie	Longfunctie en meten luchtwegontsteking bij longfunctieafdeling	X	X
		Apotheek	Inleveren en uitgifte digitale puffers	X	X
Visite 6	+/- Maand 9	Bel of video-afspraak longarts	Beoordelen effect behandeling	X	X
			Vragenlijst	X	X
		Apotheek	Bezorgen digitale puffers	X	X
Visite 7	+/- Maand 11-12	Afspraak arts-onderzoeker	Vragenlijsten	X	X
			Stop digitale puffer en digitale omgeving (Gezondheidsmeter)		
		Longfunctie	Longfunctie en meten luchtwegontsteking bij longfunctieafdeling	X	X
		Afspraak longarts	Beoordelen effect behandeling	X	X
			Longfunctie en meten luchtwegontsteking bij longfunctieafdeling	X	X
		Apotheek	Inleveren alle digitale puffers	X	X

Duur van gebruik apparaten voor digitale metingen in de thuissituatie:

- Groep 1: Eerste beweegmeter: Cardiowatch (armband): dragen gedurende 12 weken
- Groep 1: Tweede beweegmeter: DynaPort MoveMonitor (borstband): dragen gedurende 1 week
- Groep 1: Longfunctiemeter: Spirobank: gebruik gedurende 12 weken
- Groep 1: Ontstekingsmeter: Vivatmo: gebruik gedurende 12 weken
- Groep 1 en 2: Digitale puffer: Digihaler: gebruik tot einde studie
- Groep 1: Digitale omgeving: Gezondheidsmeter van Curavista (app): gebruik tot einde studie (en eventueel daarna)

Bijlage D: toestemmingsformulier proefpersoon

EXACT@home studie: Expertise Astma COPD traject met digitale ondersteuning.

- Ik heb de informatiebrief gelezen. Ook kon ik vragen stellen. Mijn vragen zijn goed genoeg beantwoord. Ik had genoeg tijd om te beslissen of ik meedoe.
- Ik weet dat meedoen vrijwillig is. Ook weet ik dat ik op ieder moment kan beslissen om toch niet mee te doen met het onderzoek. Of om ermee te stoppen. Ik hoef dan niet te zeggen waarom ik wil stoppen.
- Ik geef de onderzoeker toestemming om mijn huisarts, vorige longarts en eigen apotheek te laten weten dat ik meedoe aan dit onderzoek.
- Ik geef de onderzoeker toestemming om informatie op te vragen bij mijn huisarts, vorige longarts en eigen apotheek over o.a. ziekenhuisopnames, medicatie voorschriften, poli -, spoedeisende hulp - en huisartsbezoeken gerelateerd aan astma van voor en tijdens het onderzoek.
- Ik geef de onderzoeker toestemming om mijn huisarts en/of specialist informatie te geven over onverwachte bevindingen uit het onderzoek die van belang zijn voor mijn gezondheid
- Ik geef de onderzoekers toestemming om mijn gegevens en resultaten te verzamelen en te gebruiken. De onderzoekers doen dit alleen om de onderzoeksvraag van dit onderzoek te beantwoorden.
- Ik weet dat voor de controle van het onderzoek sommige mensen al mijn gegevens kunnen inzien. Die mensen staan in deze informatiebrief. Ik geef deze mensen toestemming om mijn gegevens in te zien voor deze controle.
- Ik weet dat ik niet aan het onderzoek mag deelnemen als ik zwanger ben of borstvoeding geef. Ik weet dat ik de onderzoeker op de hoogte moet stellen en niet mee kan doen met de studie als ik toch zwanger wordt of borstvoeding ga geven.
- Ik weet dat er tijdens de digitale thuismetingen niet wordt gecontroleerd op afwijkende waardes door de zorgverlener of onderzoeker. Wel worden de waardes achteraf beoordeeld.
- Ik geef toestemming voor het opslaan van gecodeerde, gepseudonimiseerde gegevens van de Gezondheidsmeter, de app van de eerste beweegmeter (armband), de app van de digitale puffer en de elektronische neus op een beveiligde Europese Cloud, zodat de onderzoeker en zorgverlener de data kunnen inzien en gebruiken.
- Ik geef toestemming voor het delen van gecodeerde, gepseudonimiseerde gegevens met de producenten van de Digitale inhalator (Teva, een fabrikant die deels gevestigd is buien de EU), de elektronische neus (Breathomix) en een vragenlijst die kennis, vaardigheden en vertrouwen in het kunnen managen van de eigen gezondheid of ziekte meet (PAM, Insignia Health) voor de ontwikkeling, verbetering en het naar de markt brengen van medische hulpmiddelen en producten (o.a. vragenlijsten).

- Wilt u in de tabel hieronder ja of nee aankruisen?

Ik geef toestemming om mijn gegevens te bewaren om dit te gebruiken voor ander onderzoek, zoals in de informatiebrief staat. De gegevens worden daarvoor nog 15 jaar bewaard.	Ja ☐	Nee☐
Ik geef toestemming om mij eventueel na dit onderzoek te vragen of ik wil meedoen met een vervolgonderzoek.	Ja ☐	Nee☐

Ik wil meedoen aan dit onderzoek.

Mijn naam is (proefpersoon): ………………………………..
Handtekening: ……………………… Datum : __ / __ / __

Ik verklaar dat ik deze proefpersoon volledig heb geïnformeerd over het genoemde onderzoek.

Wordt er tijdens het onderzoek informatie bekend die die de toestemming van de proefpersoon kan beïnvloeden? Dan laat ik dit op tijd weten aan deze proefpersoon.

Naam onderzoeker (of diens vertegenwoordiger):……………………………….
Handtekening:……………………… Datum: __ / __ / __
